# Natural products targeting the MAPK signaling pathway: potential options for ulcerative colitis treatment

**DOI:** 10.3389/fphar.2025.1721584

**Published:** 2026-01-22

**Authors:** Canglin Li, Zuoqiang Li, Ya Zheng

**Affiliations:** 1 Medical Management Department, Affiliated Hospital of Shandong University of Traditional Chinese Medicine, Jinan, China; 2 Department of Traditional Chinese Medicine, Shandong Academy of Occupational Health and Occupational Medicine, Jinan, China; 3 The Second Gastroenterology Department, Affiliated Hospital of Shandong University of Traditional Chinese Medicine, Jinan, China

**Keywords:** inflammation, intestinal barrier, MAPK, natural products, ulcerative colitis

## Abstract

As a chronic, recurrent inflammatory bowel disease, ulcerative colitis (UC) poses significant clinical challenges due to its progressive intestinal mucosal damage, recurrent exacerbations of abdominal pain and diarrhea, and increased risk of colorectal cancer conversion with disease duration. Conventional medications such as aminosalicylates and glucocorticoids can control inflammation in the short term, but long-term use often leads to issues like drug resistance, gastrointestinal adverse reactions, and immunosuppression, failing to meet patients’ demand for safe, long-lasting treatment. Natural products, with their wide sources, diverse structures, and rich bioactivity, offer advantages such as multi-targeted effects, low toxicity, and minimal side effects. They demonstrate great potential in treating inflammatory diseases, providing new avenues for UC therapy. Recent studies indicate that various natural products which include flavonoids, alkaloids, terpenoids, and polyphenols can effectively suppress intestinal inflammatory responses, improve intestinal barrier function, and regulate immune balance by targeting the MAPK signaling pathway. This review, using the keywords “ulcerative colitis,” “MAPK,” and “natural products,” retrieved relevant studies from PubMed, Web of Science, and CNKI databases over the past decade. This work identified 42 studies (32 from the past 5 years and 10 from the past 12 years), revealing the mechanisms by which natural products targeting the MAPK signaling pathway function in UC treatment. It provides important theoretical and experimental foundations for developing novel UC treatment strategies based on natural medicines and lays the groundwork for subsequent clinical translation studies of natural products.

## 1 Introduction

Ulcerative colitis (UC), one of the primary subtypes of inflammatory bowel disease (IBD), is a lifelong condition characterized by chronic nonspecific inflammation of the colonic mucosa. Typical pathological features include mucosal hyperemia and edema, crypt abscess formation, and continuous ulcerative lesions, which may progress to colonic cancer in severe cases. Reports indicate a significant global increase in the disease burden of UC (Zhang et al., 2024). UC has long been considered a disease prevalent in Western industrialized nations. However, both the incidence and hospitalization rates of this condition have shown a significant upward trend in emerging industrialized economies such as China, India, and Latin American countries. As of 2023, the global prevalence of ulcerative colitis has reached approximately 5 million cases. This poses a major challenge to healthcare systems and public health management worldwide (Le Berre et al., 2023).

Furthermore, beyond typical gastrointestinal symptoms like persistent diarrhea, mucus-blood stools, and lower abdominal cramps, UC patients frequently experience multiple extraintestinal complications, including anemia, malnutrition, and musculoskeletal disorders (Wangchuk et al., 2024). These symptoms exert multidimensional negative impacts on patients’ lives, severely impairing their ability to perform daily activities and significantly reducing work efficiency. They also exert a substantial psychological toll, triggering emotional issues such as anxiety and depression.

Although the pathogenesis of UC remains incompletely understood, existing research confirms that its onset is not attributable to a single factor. Instead, it results from the interaction and combined effects of multiple factors, including genetic susceptibility, intestinal immune dysfunction, compromised intestinal barrier function, and gut microbiota imbalance (Nakase et al., 2022). In clinical management, commonly employed interventions include 5-aminosalicylate drugs, glucocorticoids, thiopurine immunosuppressants, and anti-TNF-α monoclonal antibodies. While these drugs can alleviate symptoms and induce disease remission in the short term, their long-term use presents significant limitations. For example, 5-aminosalicylates show limited efficacy for moderate-to-severe UC, glucocorticoids fail to sustain remission and may induce side effects like osteoporosis and infections, while immunosuppressants and biologics carry risks of hepatic and renal impairment, opportunistic infections, and increased lymphoma incidence (Segal et al., 2021). Therefore, identifying novel therapeutic strategies with well-defined mechanisms, high safety profiles, and sustained efficacy has become a critical scientific challenge in the field of UC.

The MAPK signaling pathway, as a crucial intracellular signaling network, plays a central role in regulating physiological and pathological processes including cell proliferation, differentiation, apoptosis, and inflammatory responses (Saleem, 2024). In recent years, extensive research has confirmed that abnormal activation of the MAPK is closely associated with the pathogenesis of UC. Studies indicate that p38MAPK phosphorylation levels are significantly elevated in colonic mucosal tissues of UC patients and in experimental colitis animal models. Its excessive activation promotes the expression and release of proinflammatory cytokines by regulating transcription factors such as NF-κB, thereby exacerbating intestinal mucosal inflammatory damage (Yan et al., 2022). Furthermore, MAPK interacts with gut microbiota metabolites (e.g., short-chain fatty acids, lipopolysaccharides) and immune cells (e.g., macrophages, Th17 cells), amplifying intestinal inflammatory responses. This suggests that targeted regulation of MAPK may represent a novel therapeutic target for UC (Zhang S. et al., 2025).

Natural products, as widely distributed bioactive substances in nature, possess unique advantages such as structural diversity, broad biological activity, and relatively low toxicity, making them a crucial source for innovative drug development. In the field of inflammatory diseases, increasing evidence indicates that natural products exert anti-inflammatory effects by regulating MAPK (Haftcheshmeh et al., 2022). Moreover, compared to chemically synthesized drugs, natural products often exhibit multi-targeted and multi-pathway effects. They not only directly regulate MAPK but also synergistically exert anti-inflammatory effects through mechanisms such as modulating the gut microbiota and enhancing antioxidant stress capacity (Larabi et al., 2020).

Based on this, this paper will summarize the pharmacological actions and molecular mechanisms of different natural products targeting MAPK for treating UC, aiming to provide theoretical basis and research directions for developing novel natural drugs for UC based on MAPK regulation.

## 2 Methods and literature search strategy

**FIGURE 1:**
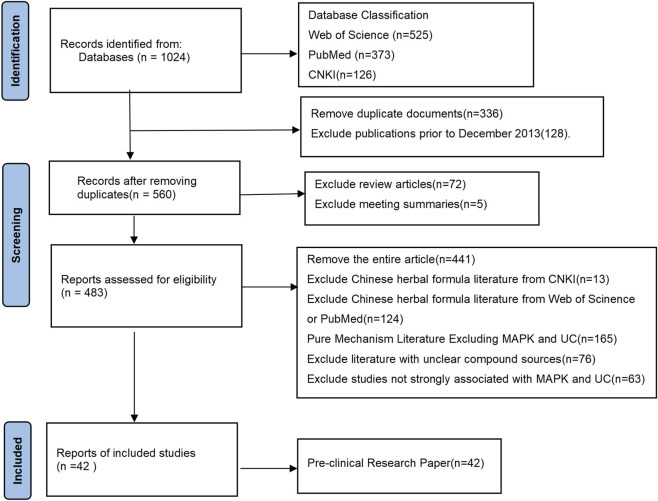
Flowchart of literature search.

This study conducted a systematic literature search across three major databases: PubMed, Web of Science, and CNKI. The search strategy employed core keywords including “MAPK”, “ulcerative colitis”, “natural products”, “Chinese herbal monomers”, “Chinese herbal extracts”, and “Chinese herbal metabolites”. These were supplemented with corresponding Medical Subject Headings (MeSH) terms and synonyms to ensure comprehensive and precise literature coverage. The initial database search yielded 1,024 articles. The timeframe for the 1,024 included studies was set from December 2013 to July 2025. Studies were screened in two sequential stages: first by title and abstract, then by full-text review. This process was conducted to identify and exclude research with significant risks of methodological bias, including, but not limited to, selection, detection, and reporting bias. Ultimately, 42 studies met the predefined inclusion criteria for this review and were included in subsequent analysis (Figure 1).

## 3 Overview of the MAPK signaling pathway

**FIGURE 2:**
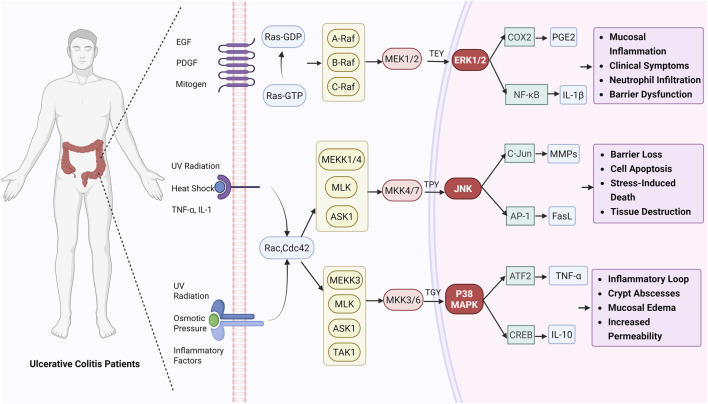
Schematic diagram of MAPK involvement in UC mechanisms.

MAPK is a highly conserved cellular signaling pathway extensively involved in regulating diverse physiological and pathological processes. The MAPK family comprises subtypes including ERK, p38 MAPK, and JNK. These proteins transmit external stimuli through a series of cascading reactions, triggering intracellular biological responses (Guo et al., 2020). MAPK plays a pivotal role in cellular processes such as proliferation, differentiation, migration, and apoptosis, mediating cellular responses to growth factors, stress signals, and changes in the extracellular environment (Arthur and Ley, 2013). Furthermore, MAPK activation is closely associated with the development of various diseases. Notably, in inflammatory responses, MAPK participates in regulating both acute and chronic inflammation by modulating immune cell activation, cytokine secretion, and the production of inflammatory mediators (Chen et al., 2023). Therefore, targeted regulation of MAPK represents a promising therapeutic strategy for inflammation-related diseases (Figure 2).

### 3.1 ERK1/2

ERK1/2 is a key member of the MAPK family in mammalian cells, serving as the terminal effector molecule in the MAPK phosphorylation cascade and widely expressed in various tissue cells. Once phosphorylated and activated, ERK can enter the nucleus to directly or indirectly regulate the expression of multiple target genes, thereby influencing cellular behavior (Martin-Vega and Cobb, 2023). Target genes activated by ERK in the nucleus regulate diverse biological processes, including cell cycle progression, differentiation, survival, and death. Pathologically, excessive ERK1/2 activation is closely linked to the onset and progression of numerous diseases, playing a particularly significant role in tumors, inflammation, and cardiovascular disorders (Schanbacher et al., 2025). In inflammatory responses, ERK1/2 serves as a pivotal signaling molecule, regulating key processes including immune cell activation, cytokine secretion, and cell migration, thereby exerting significant biological effects within the local inflammatory microenvironment (Lucas et al., 2022). Research indicates that in UC, ERK1/2 influences intestinal barrier function through its roles in immune responses, cell proliferation, and apoptosis, thereby regulating intestinal immune responses and local inflammatory states (Meng et al., 2025). Therefore, modulating ERK1/2 signaling activity may represent a potential therapeutic strategy for UC. Inhibiting excessively activated ERK1/2 signaling could potentially reduce inflammatory responses, restore intestinal barrier function, and improve clinical symptoms of the disease.

### 3.2 p38 MAPK

p38 MAPK is a key signaling molecule regulating cellular stress, inflammatory responses, apoptosis, and differentiation in eukaryotes. As a core member of the MAPK family, it comprises four p38 subunits: p38α, p38β, p38γ, and p38δ. Among these, p38α exhibits the widest distribution. Its activation depends on the “MAP3K-MAP2K-p38MAPK” three-tiered kinase cascade. In its active state, it influences the expression and synthesis of inflammation-related molecules by regulating nuclear transcription factors and cytoplasmic substrates (Coulthard et al., 2009). As a “core regulatory switch” in inflammatory responses, p38MAPK spans the entire process of inflammation initiation, progression, and resolution. It regulates the production and release of inflammatory mediators, mediates immune cell activation and infiltration, and participates in tissue damage and repair imbalance (Cuadrado and Nebreda, 2010). Research indicates that sustained p38MAPK activation is a key driver of chronic intestinal inflammation in UC. It exacerbates intestinal mucosal barrier permeability by enhancing transcription and secretion of proinflammatory factors like TNF-α and IL-1β while downregulating tight junction proteins (e.g., occludin, claudinin-1), thereby perpetuating a vicious cycle of “inflammation-barrier damage” (Ma et al., 2016). Given its central role in UC, p38MAPK inhibitors represent a potential therapeutic target. Deepening understanding of p38MAPK subtype functions may offer new avenues for precision treatment in UC.

### 3.3 JNK

JNK is a pivotal member of MAPK, playing a central role in cellular perception and response to external stimuli, as well as in regulating physiological and pathological processes. The JNK family comprises three subtypes encoded by distinct genes: JNK1 (MAPK8), JNK2 (MAPK9), and JNK3 (MAPK10). JNK1 and JNK2 are widely distributed throughout various tissues and organs, including the liver, intestines, and immune cells such as macrophages and T cells. They participate in multiple fundamental physiological and pathological processes, including systemic inflammatory responses, apoptosis, and cell proliferation (Li J. et al., 2024). Inflammation, as the body’s defensive response to injury or infection, is crucial for maintaining homeostasis when occurring appropriately. However, excessive or persistent inflammatory responses can cause tissue damage and trigger chronic inflammatory diseases. Studies indicate that activation of JNK (including elevated phosphorylation levels) is closely associated with inflammatory responses and intestinal mucosal barrier damage in UC (Yang et al., 2023). For instance, studies have revealed significantly elevated NF-κB/JNK protein levels in the colonic tissue of UC model rats, with JNK participating in regulating epithelial cell apoptosis and barrier function disruption, further impairing the repair capacity of the intestinal mucosa (Hao et al., 2022).

## 4 Mechanisms of MAPK regulation in UC

### 4.1 Suppression of inflammatory response

As a core transduction network for cellular stress responses and inflammatory reactions, dysregulation of MAPK constitutes a key molecular basis for initiating chronic inflammation, cascading amplification, and persistent tissue damage in the UC intestinal mucosa. It has emerged as a central target area for UC pathophysiological research and targeted therapy development (Foerster et al., 2022). Studies indicate that key proteins in p38MAPK (e.g., phosphorylated p38) are overexpressed in the intestinal mucosa of UC model mice, accompanied by abnormal expression of inflammatory cytokines such as IL-1β and TNF-α. These alterations correlate with disease severity, suggesting p38MAPK may participate in UC pathogenesis by regulating inflammatory responses and tissue damage (Mok et al., 2024). In UC therapy, strategies targeting MAPK signaling demonstrate significant potential. Their mechanisms extend beyond directly blocking proinflammatory signaling to include remodeling the intestinal mucosal repair microenvironment (Huang et al., 2022). Studies in UC animal models confirm that inhibiting MAPK activity significantly reduces the expression levels of proinflammatory factors such as TNF-α and IL-1β in the colonic mucosa. Concurrently, it upregulates the phosphorylation level of the epidermal growth factor receptor (EGFR), promoting the proliferation and migration of intestinal epithelial cells and accelerating the repair process of damaged mucosa (Wu et al., 2022). Furthermore, MAPK regulation can exert indirect anti-inflammatory effects by influencing gut microbiota composition and metabolic function. Studies reveal that inhibiting MAPK significantly increases the abundance of beneficial bacteria such as Bifidobacterium and *Lactobacillus* in the gut, reduces pro-inflammatory factor production, and establishes a virtuous cycle of “MAPK inhibition - optimized microbiota structure - reduced inflammation levels,” offering a new dimension for comprehensive UC treatment (Ye et al., 2024). Consequently, developing targeted MAPK regulatory strategies that suppress pro-inflammatory effects while preserving its positive role in mucosal repair represents a crucial future direction for UC treatment research.

### 4.2 Alleviating oxidative stress

Oxidative stress (OS) refers to the pathophysiological process where, under physiological or pathological conditions, the equilibrium between reactive oxygen species (ROS) and antioxidant defense systems is disrupted. This imbalance leads to excessive ROS production or diminished antioxidant capacity, ultimately causing oxidative damage (Forman and Zhang, 2021). Within the pathological microenvironment of UC, intestinal epithelial cells and immune cells undergo excessive oxidative stress, generating substantial ROS. These ROS play a pivotal role in inducing intestinal mucosal damage, promoting inflammatory responses, and disrupting barrier function (Tratenšek et al., 2024). As a key regulatory network for oxidative stress, MAPK helps cells counteract these damages through precise modulation of intracellular signaling. Elevated ROS levels activate p38MAPK, which participates in regulating multiple downstream effects related to OS, influencing the synthesis of antioxidant enzymes such as SOD and GPx (Li L. et al., 2024).

MAPK activation not only directly promotes antioxidant enzyme expression but also further enhances cellular antioxidant defense capacity through interaction with the Nrf2 signaling pathway (Ma et al., 2021). As a core regulator of the OS response, Nrf2 translocates to the nucleus upon oxidative injury, initiating transcription of antioxidant response elements (AREs) to upregulate antioxidant enzyme expression (Bellezza et al., 2018). Research indicates that the interaction between MAPK and Nrf2 provides multi-level protection for antioxidant defense in UC. During inflammatory responses, MAPK not only regulates antioxidant enzyme synthesis by activating Nrf2 but also reduces ROS production and accumulation by promoting autophagy and repairing damaged mitochondria, thereby mitigating the adverse effects of OS (Yang et al., 2022). Furthermore, MAPK effectively eliminates damaged mitochondria by regulating PINK1/Parkin-mediated mitochondrial autophagy and the ULK1 autophagy pathway, thereby reducing ROS production and preventing apoptosis, thus further mitigating OS-induced cellular damage (Foerster et al., 2022).

The above studies indicate that MAPK’s role in UC extends beyond its involvement in inflammatory responses. It also regulates OS through multifaceted mechanisms, playing a crucial regulatory role in preventing and treating UC. Therapeutic strategies targeting MAPK to modulate OS can effectively reduce colonic inflammation, repair damaged tissues, and enhance the gut’s antioxidant defense capacity, offering new insights and directions for the clinical management of UC.

### 4.3 Inhibition of apoptosis

Apoptosis is a highly regulated programmed cell death process essential for maintaining physiological equilibrium and cellular homeostasis. The apoptotic process in intestinal epithelial cells plays a critical role, particularly in inflammatory bowel diseases like UC (Wan et al., 2022). Under normal conditions, apoptosis facilitates the removal of damaged or abnormal cells, preserving intestinal ecological balance and barrier integrity. However, dysregulated apoptosis can lead to excessive death or abnormal survival of intestinal epithelial cells, thereby inducing pathological inflammatory responses and exacerbating disease progression (Li A. et al., 2023). In the pathological process of UC, the intestinal mucosal tissue is subjected to multiple stresses including persistent OS, cytokine storms, and dysbiosis of the gut microbiota. This results in the abnormal activation of apoptosis in intestinal epithelial cells. The MAPK pathway, as a key intracellular signaling network, influences the apoptotic fate of UC intestinal mucosal cells by regulating the expression and activation of downstream apoptosis-related molecules, thereby emerging as a significant potential therapeutic target for UC (Larabi et al., 2020). Reports indicate that in UC mouse models, abnormal activation of MAPK exacerbates intestinal inflammatory responses and excessive cell apoptosis. It also increases the expression of apoptosis-related proteins (such as Caspase-3 and Caspase-8), inducing intestinal epithelial cell death and thereby worsening intestinal damage (Liu C. et al., 2022).

In summary, MAPK plays a crucial role in regulating apoptosis, particularly as a key pathological mechanism in the pathogenesis and progression of UC. Targeting MAPK and its associated apoptotic factors can effectively alleviate clinical symptoms of UC and delay disease progression. Natural products offer a novel therapeutic strategy by modulating MAPK to inhibit apoptosis, providing potential targets for the clinical treatment of UC.

### 4.4 Regulation of intestinal immunity

In the chronic intestinal inflammatory pathology of UC, the MAPK family acts as a key signaling hub maintaining intestinal immune homeostasis and inflammation resolution by regulating the activation, differentiation, and cytokine secretion networks of intestinal mucosal immune cells (Wang et al., 2023a). As a vital barrier between the body and the external environment, the imbalance of the intestinal mucosal immune microenvironment (e.g., excessive infiltration of pro-inflammatory immune cells, impaired anti-inflammatory mechanisms) is a core component in UC pathogenesis. The MAPK family reshapes intestinal immune responses through multidimensional actions, providing key molecular targets for targeted UC therapy.

At the level of innate immune cells in the gut, MAPK regulates UC inflammation by modulating the functional phenotypes of macrophages and Dendritic Cells (DCs) (Zhang et al., 2022). During the acute phase of UC, intestinal macrophages exhibit excessive pro-inflammatory activation. They exacerbate mucosal damage by releasing pro-inflammatory cytokines such as TNF-α, IL-1β, and IL-6. The sustained activation of p38MAPK and JNK is the core mechanism driving this process. which phosphorylate and activate downstream transcription factors like AP-1 (e.g., c-Jun/c-Fos complexes), significantly upregulating the transcription and translation of proinflammatory cytokine genes (Yao et al., 2024). Concurrently, p38MAPK further promotes IL-1β maturation and release by regulating NLRP3 inflammasome activation in macrophages, creating a feedback loop of inflammatory amplification (Wei et al., 2021). Moreover, moderate ERK activation promotes DCs maturation and antigen presentation capacity, whereas its excessive activation inhibits DCs secretion of the anti-inflammatory cytokine IL-10. This leads to pro-inflammatory differentiation of intestinal T cells, disrupting intestinal immune tolerance and exacerbating UC progression (Miao et al., 2019).

At the therapeutic level, inhibiting p38MAPK or JNK activity significantly reduces proinflammatory cytokine levels in the intestinal mucosa of UC animal models, decreases immune cell infiltration, and improves epithelial barrier function (El-Maadawy et al., 2022). Furthermore, interventions targeting upstream regulatory molecules of MAPK (e.g., TLR4, TNF receptors) can exert therapeutic effects by indirectly inhibiting excessive MAPK activation, suggesting the feasibility and broad prospects of MAPK as a therapeutic target for UC (Ahmedy et al., 2020). However, it is important to note that the complexity of MAPK subfamily functions in intestinal physiology and pathology (e.g., the bidirectional regulatory role of ERK) suggests that future targeted therapies must achieve precise modulation of MAPK rather than simple global inhibition. This approach aims to exert anti-inflammatory effects while avoiding interference with normal intestinal immune homeostasis and epithelial repair functions.

### 4.5 Repairing the mucosal barrier

The intestinal epithelial barrier, a critical defense structure against pathogen invasion from the intestinal lumen and the spread of inflammatory mediators, is primarily composed of tight junction (TJ) complexes, the mucus layer, and subepithelial immune cells. Its structural integrity and functional stability directly determine the progression and prognosis of UC (Kaminsky et al., 2021). MAPK as a vital intracellular signaling network, exerts central regulatory roles in UC pathophysiology by modulating the functions of various components of the intestinal epithelial barrier, thereby providing key therapeutic targets for UC treatment.

At the TJ protein regulation level, MAPK dynamically modulates TJ protein expression and localization through phosphorylation mechanisms, thereby maintaining the integrity of intercellular junctions in intestinal epithelial cells (Guo et al., 2021). Within the inflammatory microenvironment of UC, proinflammatory factors such as TNF-α and IL-1β activate members of the MAPK family. Sustained activation of JNK and p38MAPK downregulates the transcriptional levels of Occludin, ZO-1, and Claudin-1 while promoting the ubiquitination and degradation of TJ proteins. This leads to loosening of epithelial cell junctions and increased intestinal epithelial permeability (Chen et al., 2018). This facilitates the entry of bacterial antigens and toxins into the submucosal layer, activating the immune system and triggering inflammation. The resulting inflammation further damages the intestinal epithelium, creating a vicious cycle that accelerates UC progression.

### 4.6 Modulating gut microbiota

The gut microbiota plays a crucial role in maintaining intestinal health. When dysbiosis occurs, beneficial bacteria decrease while pathogenic bacteria proliferate. Pathogenic bacteria secrete enterotoxins that directly damage intestinal epithelial cells and disrupt mucosal integrity. This also increases intestinal permeability, allowing pathogenic bacteria to invade and trigger a cascade of inflammatory and immune responses. Enhanced intestinal mucosal permeability facilitates the translocation of gut bacteria and their metabolic byproducts and the latter enter the enterohepatic circulation, further damaging the intestinal mucosal barrier. Additionally, abnormalities in bacterial species, abundance, and function impair the energy metabolism and immune regulatory functions of intestinal epithelial cells.

The pathological process of UC is characterized by a vicious cycle involving intestinal mucosal barrier damage, excessive immune-inflammatory activation, and dysbiosis of the gut microbiota. MAPK serves as a key transduction hub linking host immune responses to microbial metabolic signals. MAPK-mediated inflammatory responses alter the redox environment and nutritional metabolism within the gut, creating favorable conditions for pathogenic bacterial colonization and proliferation, thereby exacerbating dysbiosis. Huang et al.demonstrated that astragalin improves gut microbiota by regulating SCFAs levels, increasing butyrate and propionate content, thereby promoting colonic mucosal barrier repair and modulating gene expression related to the SIRT1/p38MAPK inflammatory pathway (Huang et al., 2022). Lei et al.demonstrated that targeting MAPK (particularly p38) modulates *Clostridium difficile* expression. This approach exerts its effects by suppressing key pro-inflammatory factors (e.g., IL-6 and TNF-α), thereby blocking inflammatory cascades while simultaneously promoting gut microbiota homeostasis and intestinal barrier repair. This reverses microbiota-mediated treatment resistance and effectively improves UC disease severity (Lei et al., 2025). Additionally, Zang et al.demonstrated that *Candida* utilis acts through multiple mechanisms to alleviate UC. By elevating beneficial bacteria (e.g., Prevotellaceae, *Lactobacillus*) and suppressing pathogens (e.g., Bacteroidetes), it restores gut microbiota diversity and SCFA levels. Concurrently, it inhibits the NF-κB/MAPK pathway, decreases pro-inflammatory factors (e.g., IL-1β), mitigates oxidative stress, repairs the intestinal barrier, and reduces symptoms (Zang et al., 2025). These studies indicate that by modulating the host’s intestinal metabolic environment and promoting the enrichment of SCFAs-producing microbiota, a stable ecological niche is provided for probiotics, forming a positive regulatory cycle of “MAPK inhibition-microbiota restoration-inflammation alleviation”. Therefore, regulating and maintaining microbial balance holds potential therapeutic significance for UC.

## 5 Targeted MAPK therapy with natural products

**FIGURE 3:**
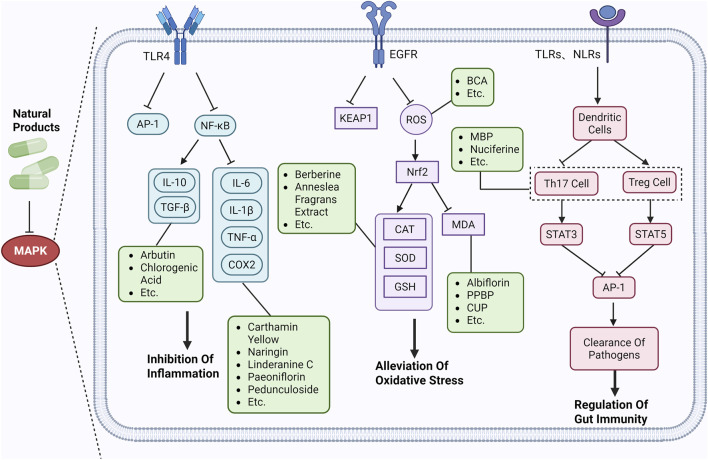
Mechanisms by which natural products target MAPK to treat UC through inhibiting inflammation, alleviating oxidative stress, and regulating gut immunity.

**FIGURE 4:**
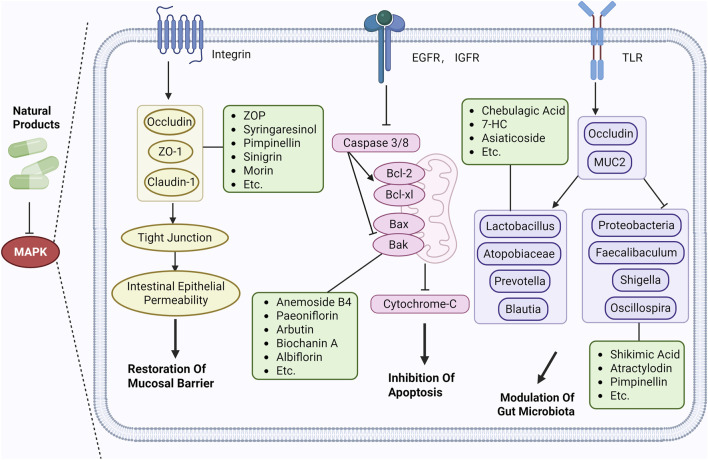
Mechanisms by which natural products target MAPK to treat UC through repairing the mucosal barrier, inhibiting apoptosis, and modulating the gut microbiota.

Extensive research indicates that natural products (such as flavonoids, terpenoids, alkaloids, and polyphenols) regulate intestinal mucosal barrier function via MAPK. This action reduces inflammatory infiltration in the gut, improves histopathological damage to intestinal tissues, decreases the release of pro-inflammatory cytokines, inhibits excessive apoptosis of intestinal epithelial cells, and enhances the gut’s antioxidant stress capacity. These products demonstrate significant therapeutic potential for treating UC. As shown in (Table 1; Figures 3, 4).

**TABLE 1:** Natural products regulate MAPK signaling pathway to treat ulcerative colitis.

Extract	Origination	Structure	Model	Biological effects	Results	References
Chlorogenic acid	*Lonicera japonica Thunb*	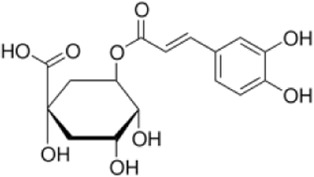	C57BL/6 mice	CMDI↓TNF-α↓IL-1β↓IL-6↓ERK1/2↓p38MAPK↓JNK↓MPO↓SOD↑IL-10↑	Inhibiting MAPK activation to mitigate colonic tissue damage and improve oxidative stress status	Gao et al.(2019)
Ellagic acid	*Punica granatum L*	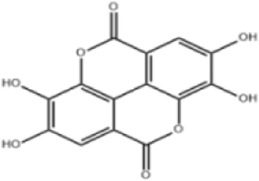	C57BL/6 mice	DAI↓COX-2↓iNOS↓p38 MAPK↓NF-κB↓	Inhibit the inflammatory response	Marín et al.(2013)
Biochanin A	*Trifolium pratense L*	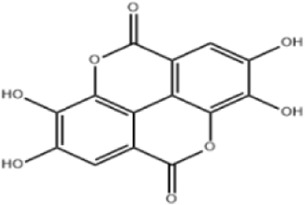	RAW264.7 cells C57BL/6 mice	ROS↓TNF-α↓IL-1β↓IL-18↓ MAPK(p38、JNK、ERK)↓ GSH↑SOD↑	Inhibit the inflammatory response Improve the oxidative stress status	Kulhari et al.(2024)
Morin	*Styphnolobium japonicum (L.) Schott*	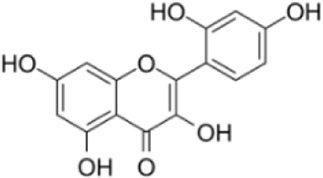	C57BL/6 mice	MAPK/NF-κB↓IL-6↓IL-1β↓TNF-α↓Claudin-3↑Occludin↑ ZO-1↑	Suppress inflammatory responses and maintain intestinal barrier integrity	Qiu et al.(2024)
Carthamin yellow	*Carthamus tinctorius L*	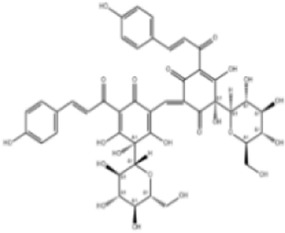	Caco-2 cell C57BL/6 mice	TNF-α↓IL-6↓IL-1β↓ MAPK/NF-κB↓	Suppressing inflammatory responses and improving oxidative stress	Bian et al.(2024)
Naringin	*Citrus limon (L.) Osbeck*	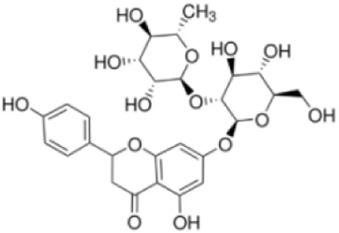	RAW264.7 cells C57BL/6 mice	NF-κB↓TNF-α↓IL-1β↓IL-6↓ MAPK↓	Suppress inflammatory responses and reduce damage to colonic tissue	Cao et al.(2018)
Fisetin	*Fragaria vesca L*	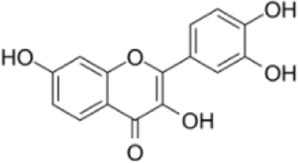	Balb/C mice	TNF-α↓IL-1β↓IL-6↓iNOS↓ p38 MAPK↓GSH↑	Inhibit the inflammatory response Improve weight loss	Sahu et al.(2016)
Kushenol I	*Sophora flavescens Ait*	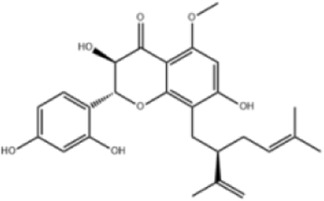	C57BL/6 mice	p-p38 MAPK↓IL-1β↓IL-6↓ IL-17↓TNF-α↓IL-10↑GSH-PX↑SOD↑	Suppress oxidative stress, regulate immune function, and maintain intestinal barrier integrity	He et al.(2025)
Linderanine C	*Lindera aggregata (Sims) Kosterm*	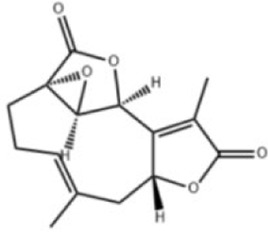	RAW264.7 cells C57BL/6 mice	IL-6↓IL-1β↓TNF-α↓IL-6↓ERK↓p38MAPK↓	Inhibit the inflammatory response	Lan et al. (2024)
Albiflorin	*Nigella sativa L*	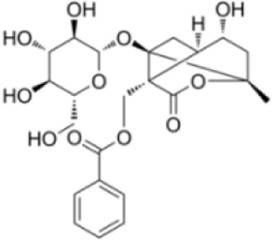	C57BL/6 mice	MPO↓MDA↓TNF-α↓IL-6↓ IL-1β↓MAPK↓SOD↑GSH↑	Suppressing inflammatory responses and regulating immune function	Wang et al.(2022)
Bilobalide	*Ginkgo biloba L*	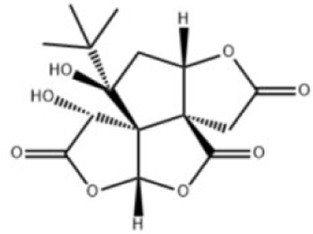	C57BL/6 mice	IL-1β↓IL-6↓TNF-α↓MAPK↓ ZO-1↑Occludin↑Claudin-3↑	Maintain the integrity of the intestinal barrier	Zhang Y. et al.(2021)
Oleanolic acid 28-O-β-D-glucopyranoside	*Panax ginseng C.A.Mey*	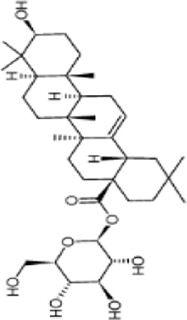	Balb/C mice Caco-2 cell	MAPK↓MDA↓MPO↓IL-6↓TNF-α↓COX-2↓iNOS↓SOD↑GSH↑ZO-1↑Occludin↑Claudin-1↑	Regulate immune function and suppress oxidative stress	Wang J. et al.(2024)
Anemoside B4	*Pulsatilla chinensis (Bunge) Regel*	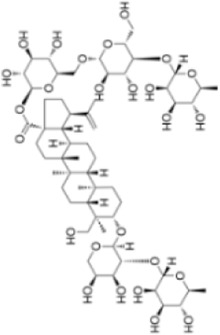	Sprague-Dawley rats	S100A9↓leaved-caspase 3↓p53↓ MAPK↓IL-1β↓IL-6↓TNF-α↓ Bcl-2/Bax↓	Inhibit the inflammatory response	Zhang H. et al.(2021)
Paeoniflorin	*Paeonia lactiflora Pall*	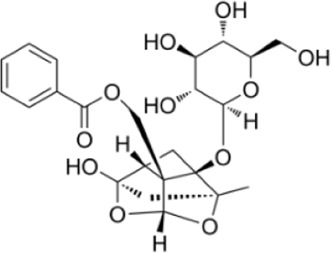	Balb/C mice	NF-κB↓IL-1β↓IL-6↓p38MAPK↓	Inhibit the inflammatory response Inhibit cell apoptosis	Gu et al.(2017)
Pedunculoside	*Paeonia lactiflora Pall*	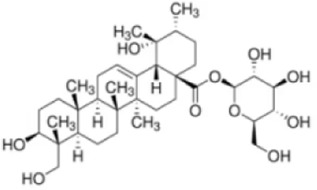	RAW264.7 cells C57BL/6 mice	IL-1β↓IL-6↓TNF-α↓COX-2↓iNOS↓ERK1/2↓JNK1/2↓P38↓	Improve the pathological structure of colonic tissue	Liu et al.(2020)
Asiaticoside	*Centella asiatica (L.) Urb*	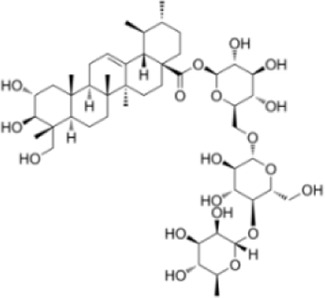	C57BL/6 mice	MAPK↓IL-1β↓IL-6↓TNF-α↓MPO↓COX-2↓iNOS↓↓ claudin-3↑occludin↑ZO-1↑	Maintain the integrity of the intestinal barrier	Liu et al.(2023)
α-Bisabolol	*Matricaria chamomilla L*	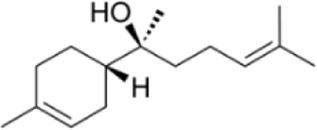	C57BL/6 mice HT-29 cell	MAPK↓IL-1β↓IL-6↓TNF-α↓	Inhibit the inflammatory response	Venkataraman et al.(2022)
Stevioside	*Matricaria chamomilla L*	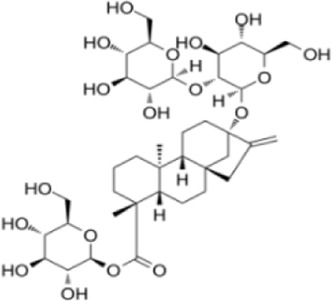	RAW264.7 cell Balb/C mice	DAI↓p38MAPK↓JNK↓TNF-α↓ SOD↑CAT↑GSH↑	Improve the pathological structure of colonic tissue	Alavala et al.(2019)
Nuciferine	*Nelumbo nucifera Gaertn*	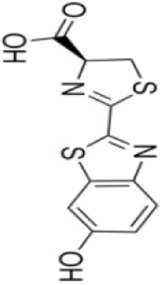	RAW264.7 cell Balb/C mice	TNF-α↓IL-1β↓p38MAPK↓JNK↓	Inhibit the inflammatory response	Kulhari et al.(2023)
Berberine	*Coptis chinensis Franch*	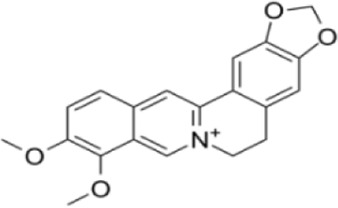	Wistar rats	TNF-α↓IL-1β↓IL-6↓MPO↓ SOD↓MDA↓NO↓p38MAPK↓GSH↑iNOS↑	Inhibit oxidative stress	Jia et al.(2020)
Mesona chinensis Benth polysaccharides	*Platostoma palustre (Blume) A.J.Paton*	—	C57/BL6 mice	DAI↓TNF-α↓IL-1β↓MAPK↓MDA↓ZO-1↑Occludin↑MUC-2↑ *Lactobacillus*↑Coprococcus↑	Improve the pathological structure of colon tissue and regulate the gut microbiota	Lu et al.(2022)
Pyrus pashia Buch polysaccharide	*Pyrus ussuriensis Maxim*	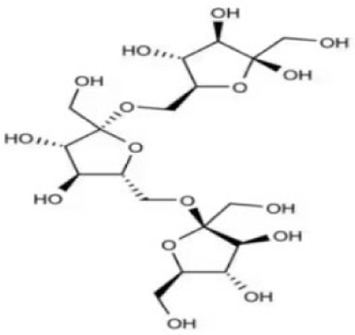	C57/BL6 mice	TNF-α↓IL-6↓IL-1β↓iNOS↓MDA↓MPO↓p38MAPK↓	Maintain the integrity of the intestinal barrier	Zhang S. et al.(2025)
Zingiber officinale polysaccharide	*Zingiber officinale Roscoe*	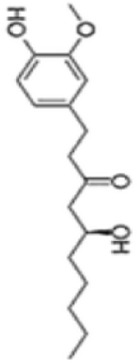	C57/BL6 mice	TNF-α↓IL-6↓IL-1β↓MDA↓MAPK↓TLR4/MyD88/NF-κB↓SOD↑Occludin↑ZO-1↑	Alleviate damage to the intestinal mucosa and inhibit oxidative stress	Jing Y. et al.(2025)
Polysaccharides of C. nudiflora Hook	*Callicarpa nudiflora Hook. and Arn*	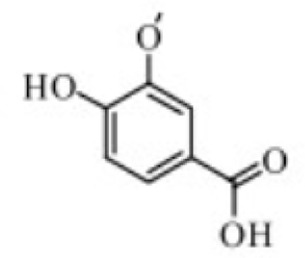	Balb/C mice	DAI↓MPO↓MDA↓TNF-α↓ IL-6↓IL-1β↓p38MAPK↓NF-κB p65↓JNK↓MUC-2↑	Inhibit the inflammatory response	Feng et al.(2024)
Shikimic acid	*Illicium verum Hook.f*	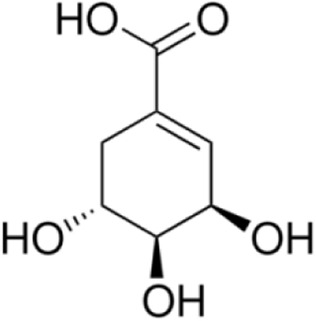	C57BL/6J mice	DAI↓NF-κB/MAPK↓TNF-α↓IL-1β↓IFN-γ↓Bacteroidetes↑Proteobacteria↓	Maintain intestinal barrier integrity and regulate gut microbiota	Li X. et al.(2023)
N-Acetyldopamine Dimer	*Isaria cicadae Miquel*	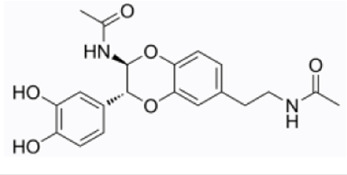	RAW264.7 cells C57BL/6 mice	DAI↓NF-κB/MAPK↓TNF-α↓IL-1β↓ERK↓	Inhibit the inflammatory response	Huang et al.(2022)
7-hydroxycoumarin	*Phaseolus vulgaris L*	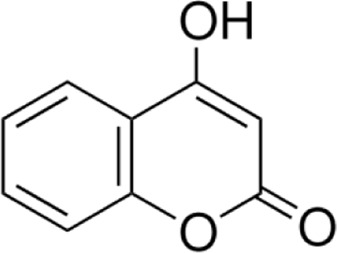	male ICR mice RAW264.7 cell	MAPK↓TNF-α↓IL-1β↓ERK↓Faecalibaculum↓*Lactobacillus*↑	Suppressing oxidative stress and regulating the gut microbiota	Liu M. et al.(2024)
Amauroderma rugosum	*Ganodermataceae*	—	RAW264.7 cell Balb/C mice	DAI↓MAPK↓NO↓TNF-α↓ IL-1β↓TNF-α↓IL-6↓IL-1β↓	Regulate immune function and improve colonic tissue pathology regulating immune function and improving colonic tissue pathology	Li J. et al.(2024)
Extract of Atractylodis Rhizoma	*Atractylodes lancea (Thunb.) DC.*	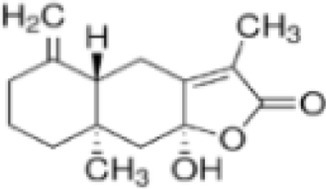	RAW264.7 cell Balb/C mice	MAPK↓NO↓TNF-α↓IL-1β↓TNF-α↓IL-6↓IL-1β↓JNK↓ERK↓	Inhibit the inflammatory response	Lin et al.(2022)
Citrus unshiu peel water extract	*Citrus aurantium f. deliciosa (Ten.) M.Hiroe*	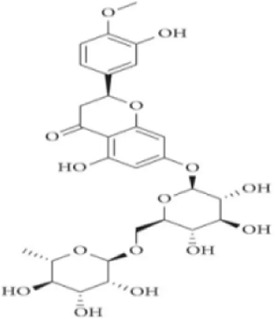	Balb/C mice	ROS↓MDA↓MPO↓PI3K/Akt↓ p38MAPK↓c-Fos↓c-Jun↓TNF-α↓IL-6↓IL-1β↓	Improve the pathological structure of colonic tissue	Lee et al.(2022)
Anneslea fragrans extract	*Anneslea fragrans Wall*	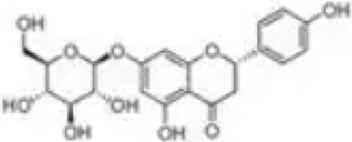	RAW264.7 cells C57BL/6 mice	NF-κB↓MAPK↓IκBα↓ZO-1↑Occludin↑Claudin-1↑TNF-α↓IL-1β↓IL-6↓IL-4↓IL-10↑ SOD↑GSH↑	Suppress inflammation and oxidative stress	Deng et al.(2021)
Flower extract of Caragana sinica	*Caragana sinica (Buc’hoz) Rehder*	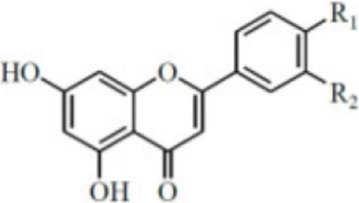	C57BL/6 mice	TLR4/MAPK↓TNF-α↓IL-1β↓ IL-6↓p-ERK↓IL-10↑SOD↓ CAT↑GSH↑TLR4↓MyD88↓	Reduce the degree of colonic lesions Regulate immune function	Li et al.(2021)
Syringaresinol	*Dracaena draco (L.) L*	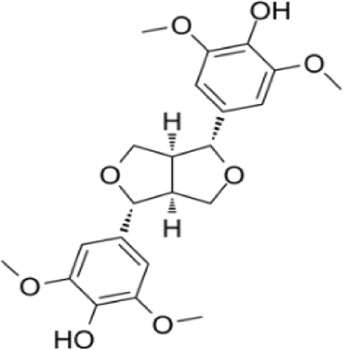	Balb/C mice Caco-2 cell	ZO-1↑occludin↑claudin-1↑E-cadherin↑TNF-α↓IL-6↓IFN-γ↓ COX-2↓MAPK↓↓GSK-3β↓β-catenin↓ E-cadherin↑	Maintain intestinal barrier integrity and improve colonic histopathology	Liu K. et al.(2024)
Pimpinellin	*Zanthoxylum asiaticum (L.) Appelhans, Groppo and J.Wen*	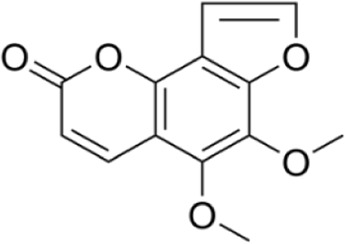	C57BL/6 mice	DAI↓ZO-1↑occludin↑claudin-3↑ TNF-α↓IL-1β↓IL-6↓p-p38↓p-JNK↓p-ERK↓ Lactobacillaceae、S24-7↑ Enterobacteriaceae、*Shigella*↓	Improve weight loss Improve the pathological structure of colonic tissue Regulate the intestinal flora	Lv et al.(2025)
Sinigrin	*Brassica oleracea L*	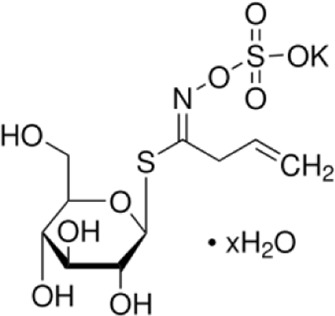	C57BL/6 mice	occludin↑claudin-3↑TNF-α↓ IL-1β↓IL-6↓p38MAPK↓JNK↓ERK↓	Inhibit the inflammatory response	Kotipalli et al.(2023)
Osthole	*Cnidium monnieri (L.) Cusson*	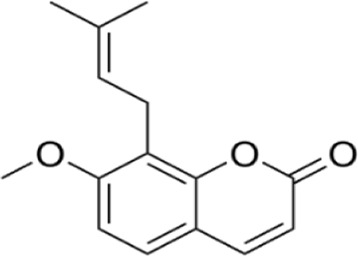	RAW264.7 cell Balb/C mice	DAI↓ TNF-α↓NF-κB↓ p38 MAPK↓NO↓PGE2↓TNF-α↓	Improve the pathological structure of colonic tissue	Fan et al.(2019)
Phillygenin	*Forsythia suspensa (Thunb.) Vahl*	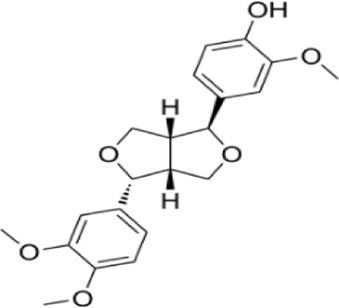	RAW264.7 cell Balb/C mice	MDA↓TNF-α↓IL-1β↓IL-6↓ MPO↓TLR4↓↓p38MAPK↓ JNK↓ NF-κB↓	Inhibit the inflammatory response	Xue et al.(2023)
Arbutin	*Arctostaphylos uva-ursi (L.) Spreng*	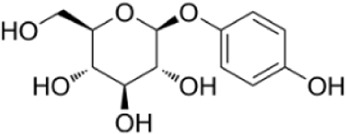	BALB/c mice	TNF-α↓↓IL-6↓IL-10↑p-JNK↓p38MAPK↓	Improve the pathological structure of colonic tissue	Zhang Y. et al.(2021)
Atractylodin	*Atractylodes macrocephala Koidz*	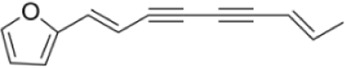	RAW264.7 cell Balb/C mice	MAPK↓TNF-α↓IL-6↓IL-1β↓iNOS↓GAPDH↓TNF-α↓Akkermansia↑Alistipes↑Desulfovibrio↓	Inhibit the inflammatory response Regulate the intestinal flora	Qu et al.(2021)
Alliin	*Allium sativum L*	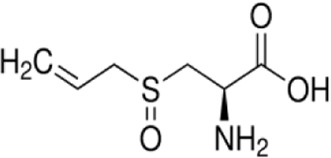	ICR mice RAW264.7 cell	MDA↓NO↓TNF-α↓IL-1β↓IL-6↓iNOS↓p38MAPK↓JNK↓ERK1/2↓NF-κB↓	Improve the pathological structure of colonic tissue	Shi et al.(2017)
Octacosanol	*Saccharum × sinense Roxb*	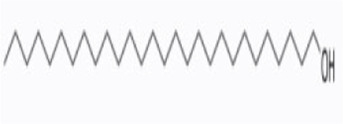	ICR mice RAW264.7 cell	TNF-α↓IL-1β↓IL-6↓iNOS↓ MDA↓c-Jun↓NF-κB↓p38MAPK↓ JNK↓ERK↓	Inhibit the inflammatory response	Guo et al.(2017)
Chebulagic acid	*Terminalia chebula Retz*	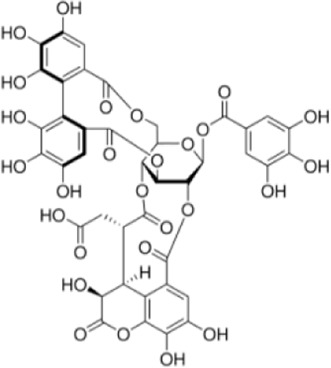	C57BL/6 mice	SOD↑GSH-PX↑MDA↓MAPK↓ NRF2/HO-1↑TNF-α↓IL-6↓ IL-1β↓ZO-1↑Occludin↑*Clostridium*_sensu_stricto_1↓ Faecalibacterium↑Dubosiella↑Muribaculaceae↑	Improve the pathological structure of colonic tissue and maintain the integrity of the intestinal barrier	Zhang et al.(2025a)

### 5.1 Phenolic products

Phenolic products, ubiquitous natural bioactive substances in the plant kingdom, derive their unique pharmacological effects primarily from the chemical reactivity of their phenolic hydroxyl (-OH) groups and the configuration of substituents. Notably, these products exhibit diverse properties including anti-inflammatory, antioxidant, and antitumor activities, demonstrating significant therapeutic potential in UC treatment.

Chlorogenic acid (CGA), a phenolic compound primarily found in plants of the Eucommiaceae like *Lonicera japonica Thunb.*, exhibits multifaceted biological activities including antioxidant, anti-inflammatory, antibacterial, and immunomodulatory effects (Nguyen et al., 2024). Gao et al. Established DSS-induced UC mouse models to investigate CGA’s therapeutic effects and potential mechanisms. Experimental results demonstrated that high-dose CGA (120 mg/kg) significantly reduced CMDI scores, alleviated colonic tissue damage, suppressed expression of inflammatory factors TNF-α, IL-1β, and IL-6 in intestinal tissues, elevated levels of the anti-inflammatory factor IL-10, and improved oxidative stress status (decreased PGE2 and MPO, increased SOD). Its efficacy was comparable to that of SASP (100 mg/kg). These findings suggest that CGA’s regulation of MAPK, leading to alterations in intestinal inflammatory factor expression, may represent one of the key mechanisms underlying its anti-UC effects (Gao et al., 2019). However, this study was conducted solely in animal models and did not include human clinical trials or pharmacokinetic analysis. Further research is required to validate its efficacy, safety, and mechanism of action in humans.

Ellagic acid (EA) is a natural polyphenolic product widely present in plants such as pomegranate (*Punica granatum L*.), exhibiting potent antioxidant and anti-inflammatory activities (Zhang et al., 2023). Experimental results showed that in acute models, although EA did not significantly improve colon weight/length ratio or MPO activity, it markedly reduced levels of pro-inflammatory cytokines (IL-6, TNF-α, IFN-γ) in colon tissue and downregulated COX-2 and inducible iNOS expression. In the chronic model, EA significantly reduced DAI scores and colon weight/length ratios, alleviated intestinal mucosal ulcers and inflammatory cell infiltration, while effectively inhibiting abnormal COX-2 and iNOS expression, blocking p38 MAPK phosphorylation, suppressing IκBα phosphorylation and p65 subunit nuclear translocation in NF-κB, and downregulating IL-6/STAT3 activation, suggesting that EA may exert gut-protective effects by synergistically inhibiting inflammatory responses through multiple signaling pathways (Marín et al., 2013). Unfortunately, while this study clarified EA’s synergistic anti-inflammatory effects through regulating multiple key inflammatory pathways including p38 MAPK, NF-κB, and IL-6/STAT3, it did not further validate the cross-talk mechanisms between these pathways. It also failed to explore EA’s potential roles in gut microbiota regulation or mucosal barrier repair, and did not establish the minimum effective dose or long-term safety profile of EA.

### 5.2 Flavonoids

Flavonoids are a class of natural organic products widely distributed in plants, primarily found in various plant parts such as fruits, leaves, petals, and seeds. These products exhibit remarkable and diverse biological activities due to their unique C6-C3-C6 basic molecular skeleton and functional group modifications at different positions, such as hydroxyl, methyl, and glycosidic groups. These activities include antioxidant effects, free radical scavenging, immune regulation, inhibition of tumor cell proliferation, anti-inflammatory and analgesic properties, as well as antibacterial and antiviral actions.

As an isoflavone product with estrogenic activity, Biochanin A (BCA) is naturally occurring in red clover and various other leguminous plants rather than being synthetically produced. It exhibits pharmacological properties including antioxidant, anti-inflammatory, and anti-apoptotic effects (Li X. et al., 2024). In a recent study, Kulhari et al. Employed LPS-induced RAW264.7 macrophage models and DSS-induced mouse UC models to evaluate BCA’s inhibitory effects on inflammatory responses and its potential mechanisms. *In vitro* experiments demonstrated that BCA (15–60 µM) significantly suppressed the release of proinflammatory cytokines such as TNF-α, IL-1β, and IL-18 induced by LPS, while inhibiting the protein expression of iNOS and COX-2, without exhibiting significant cytotoxicity. Further animal studies demonstrated that oral administration of BCA (20, 40 mg/kg) effectively alleviated DSS-induced weight loss, colon shortening, and tissue structural damage, while improving colonoscopy and histological scores. This may be associated with the ability of BCA to attenuate oxidative stress injury by inhibiting the phosphorylation of MAPK and NF-κB (p65), downregulating the expression of pro-inflammatory cytokines, upregulating the levels of GSH and SOD, and reducing the MDA content (Kulhari et al., 2024). Therefore, BCA may represent a promising therapeutic option for anti-UC effects by targeting MAPK/NF-κB and oxidative stress. However, this study has limitations: long-term drug administration or high-dose toxicity and safety were not investigated, and chronic toxicity studies are still required before clinical translation.

Morin is a natural phytochemical found in *Styphnolobium japonicum (L.) Schott*, exhibiting anti-inflammatory, antioxidant, antitumor, and neuroprotective effects (Caselli et al., 2016). To investigate Morin’s anti-UC mechanism, Qiu et al. treated DSS-induced UC mouse models with Morin. Results demonstrated that Morin (10, 20, 40 mg/kg) significantly improved weight loss, shortened colon length, and reduced DAI scores. It alleviated inflammation by inhibiting the release of downstream inflammatory factors IL-6, IL-1β, and TNF-α in MAPK/NF-κB. Furthermore, Morin upregulated the expression of tight junction proteins Claudin-3, Occludin, and ZO-1, maintaining intestinal barrier integrity, increasing goblet cell numbers, and reducing tissue damage and neutrophil infiltration. Gut microbiota analysis revealed that Morin intervention restored the intestinal microbiome structure, with increased abundance of probiotic bacteria such as Muribaculaceae and Erysipelotrichaceae and decreased abundance of harmful bacteria (Qiu et al., 2024). Thus, Morin’s regulation of MAPK/NF-κB may represent a key mechanism against UC and its risk factors. However, this study has limitations. Although Morin’s low *in vivo* bioavailability has been reported, tissue or plasma concentrations were not measured, lacking pharmacokinetic support.

Carthamin yellow (CY) is a water-soluble flavonoid pigment extracted from the *Carthamus tinctorius L.*, exhibiting potent anti-inflammatory and antioxidant pharmacological properties (Lu et al., 2019). Bian et al. Employed DSS-induced UC mouse models to elucidate CY’s potential therapeutic effects and molecular mechanisms. Experimental results demonstrated that CY (20, 40 mg/kg) significantly improved UC-related clinical manifestations, including weight loss, elevated DAI scores, and shortened colon length. CY treatment markedly reduced the expression of proinflammatory factors TNF-α, IL-6, and IL-1β, mitigating pathological inflammatory responses in colonic tissue by inhibiting MAPK/NF-κB. Furthermore, CY enhanced intestinal barrier function by upregulating the expression of ZO-1, Occludin, Claudin-1, and mucin MUC2. *In vitro* experiments using a Caco-2 cell model of inflammatory injury also demonstrated CY’s protective effects (Bian et al., 2024). This study provides valuable insights into the potential therapeutic application of CY in the prevention and treatment of UC. In the future, CY may emerge as a promising novel therapeutic candidate for UC.

Naringin, a common flavonoid compound in citrus fruits, is primarily found in the fruit of Citrus grandis and in the peel and pulp of Citrus paradisi such as *Citrus limon (L.) Osbeck*. It exhibits significant anti-inflammatory, antioxidant, and tissue-protective effects (Shilpa et al., 2023). Experiments by Cao et al. demonstrated that naringin (25, 50, 100 mg/kg) improved colon shortening and reduced histological damage in UC mice model. Further research revealed that naringin significantly reduced the expression of inflammatory cytokines TNF-α, IL-1β, and IL-6, inhibited activation of MAPK and NLRP3 inflammasome, and regulated the expression of tight junction protein ZO-1, thereby maintaining intestinal barrier integrity (Cao et al., 2018). These findings suggest naringin may serve as a natural anti-inflammatory candidate drug with clinical translation potential, playing a significant role in UC adjunctive therapy. However, the study has limitations, such as not investigating naringin’s pharmacokinetic properties, stability, or its metabolic processes within the gut microbiota which could impact its clinical application.

Fisetin is a natural plant flavonoid widely present in various fruits, vegetables, and plants like *Fragaria vesca L*. It has garnered research attention for its potential health benefits, particularly in antioxidant, anti-inflammatory, and anti-cancer activities (Kashyap et al., 2019). One study investigated the protective mechanisms of fisetin in DSS-induced acute colitis mouse models. Results demonstrated that fisetin (5, 10 mg/kg) significantly alleviated colitis symptoms, manifested as reduced DAI scores, mitigated weight loss, and decreased shortening of colon length. Additionally, fisetin effectively restored tissue GSH levels and reduced MDA accumulation, suggesting antioxidant effects. At the histological level, fisetin improved DSS-induced destruction of crypt architecture and inflammatory cell infiltration. Regarding anti-inflammatory mechanisms, fisetin significantly inhibited activation of NF-κB by reducing IκBα phosphorylation and degradation, thereby decreasing NF-κB (p65) nuclear translocation and DNA-binding activity. Concurrently, fisetin downregulated the expression of proinflammatory factors TNF-α, IL-1β, IL-6, COX-2, and iNOS, while inhibiting the activation of Akt and p38 MAPK (Sahu et al., 2016). These findings suggest that fisetin exerts anti-inflammatory and antioxidant effects through multi-pathway coordinated intervention, thereby significantly ameliorating inflammatory manifestations in experimental colitis.

Kushenol I (KSCI) is a naturally occurring isoflavone compound primarily derived from *Sophora flavescens Ait.* of the Fabaceae family, which exhibits potent anti-inflammatory, antioxidant, and antitumor effects. Research has demonstrated that KSCI exerts anti-UC effects by significantly modulating the gut microbiota and protecting intestinal barrier integrity (Chen et al., 2020). In DSS-induced UC mouse models, oral administration of KSCI (50–100 mg/kg) and the positive control drug SASP (703 mg/kg) both effectively mitigated weight loss, improved colon shortening, and reduced DAI, suggesting its potential efficacy in alleviating UC phenotypes. Mechanistic studies revealed that KSCI suppresses intestinal immune inflammation by inhibiting the activation of inflammatory pathways including p-PI3K, p-AKT, p-p38 MAPK, and NF-κB p-p65. This leads to reduced expression of proinflammatory cytokines such as IL-1β, IL-6, IL-17, and TNF-α, while upregulating the anti-inflammatory cytokine IL-10. Concurrently, KSCI significantly increased GSH-PX and SOD activity while decreasing MDA and MPO levels, indicating its capacity to alleviate OS. Notably, KSCI restored the expression of tight junction proteins ZO-1 and Occludin, repaired mucosal structure, and reduced serum LPS levels, suggesting its crucial role in maintaining the intestinal mucosal barrier (He et al., 2025). Therefore, employing KSCI to counteract inflammatory damage represents an effective strategy for protecting against intestinal mucosal injury. However, this study did not evaluate long-term toxicity or metabolic stability, limiting its ability to conclusively demonstrate KSCI’s safety for chronic disease intervention.

### 5.3 Terpenoids

Terpenoids are a class of natural organic products with extremely widespread distribution in nature, particularly concentrated in secretory tissues of plant organs such as leaves, stems, roots, flowers, and fruits. These products exhibit remarkable and diverse biological activities due to their molecular frameworks formed by the polymerization of diverse isoprene units and their association with various oxygen-containing functional groups (e.g., hydroxyl, carbonyl, ester groups). These activities include antibacterial and anti-inflammatory effects, regulation of nervous system function, antitumor properties, lipid-lowering effects, antioxidant activity, and aromatic properties as fragrance components, among other important pharmacological and physiological functions.

Linderanine C (LDC) is a representative sesquiterpene lactone natural compound extracted from *Lindera aggregata (Sims) Kosterm.*, exhibiting anti-inflammatory and antioxidant pharmacological effects. In DSS-induced UC mouse models, both LDC (6, 20 mg/kg) and SASP (200 mg/kg) reduced DAI scores, improved colonic histopathological damage, and mitigated mucosal structural disarray. This mechanism is associated with inhibition of MAPK. *In vitro* studies further revealed that LDC effectively suppressed LPS-induced M1 polarization in RAW264.7 macrophages at concentrations ≤400 μmol/L without significant cytotoxicity. It reduced expression of the M1 marker CD86 and markedly decreased secretion of inflammatory mediators TNF-α, IL-6, and NO. In summary, these effects may stem from LDC’s ability to inhibit GPCR-mediated MAPK, manifested as reduced phosphorylation levels of ERK, p38, and JNK, demonstrating multi-target anti-inflammatory properties (Lan et al., 2024). However, further pharmacokinetic, toxicity, and drug-drug interaction studies are required before clinical application of LDC. Future research should focus on developing targeted delivery systems and exploring multi-omics-based target networks.

Albiflorin is a monoterpenoid glycoside compound extracted from the dried roots of plants *Nigella sativa L.* in the Ranunculaceae family. Modern pharmacological studies indicate that Albiflorin possesses anti-inflammatory, antioxidant, and anti-apoptotic properties (Ou et al., 2024). Wang et al. investigated the effects of Albiflorin on DSS-induced UC model mice and its underlying mechanisms. Following treatment with Albiflorin (50, 100 mg/kg), mice exhibited significant improvements in weight loss, colon shortening, and histopathological damage. Levels of OS and inflammatory markers which including MPO, MDA, TNF-α, IL-6, and IL-1β, were markedly reduced, while SOD and GSH activities were enhanced. Furthermore, Albiflorin markedly inhibited activation of NF-κB and MAPK, including decreased phosphorylation levels of p65, p-IκBα, p-p38, p-ERK, and p-JNK (Wang et al., 2023b). These findings suggest that Albiflorin may regulate immune tolerance by inhibiting NF-κB and MAPK, alleviating OS and inflammatory responses, ultimately improving clinical and histological symptoms of UC. However, this study has limitations, such as the lack of clarification on Albiflorin’s metabolic characteristics, bioavailability, and safety margin *in vivo*, which restricts its foundational support for clinical translation.

Bilobalide (BI) is a sesquiterpene lactone compound isolated from *Ginkgo biloba L.* which has potent anti-UC effects through multi-targeted, multi-mechanistic actions (Lu et al., 2021). In DSS-induced UC mouse models, BI treatment (2.5, 5, 10 mg/kg) significantly improved colonic histopathology and mitigated weight loss. Concurrently, BI upregulates ZO-1, Occludin, and Claudin-3 expression, restoring intestinal epithelial barrier integrity. It also downregulated proinflammatory cytokine expression (IL-1β, IL-6, TNF-α) in colonic tissue and inhibited phosphorylation of AKT/NF-κB and MAPK (including p38, JNK, ERK), thereby suppressing proinflammatory factor expression and exerting anti-inflammatory effects. Furthermore, in regulating the gut microbiota, BI increased the relative abundance of probiotic *Lactobacillus* and Firmicutes while reducing the proportion of Dubosella and Bacteroidetes, improving dysbiosis and further synergistically alleviating intestinal inflammatory responses (Zhang Y. et al., 2021). However, this study remains relatively limited, lacking systematic comparisons with existing first-line therapeutic agents such as SASP and 5-ASA, and thus does not establish its clinical reference value.

Oleanolic acid 28-O-β-D-glucopyranoside (OAG) is a pentacyclic triterpenoid compound isolated from *Panax ginseng C.A.Mey.* Which could suppress pro-inflammatory factor expression and modulates OS levels. In LPS-induced Caco-2 cell models, OAG reduced IL-6, TNF-α, COX-2, and iNOS expression while enhancing ZO-1, Occludin, and Claudin-1 expression and localization, demonstrating protective effects on the intestinal barrier. In DSS-induced UC mouse models, OAG demonstrated significant anti-inflammatory and antioxidant effects by reducing DAI scores, restoring colon length, mitigating histological damage, decreasing pro-inflammatory factor levels, enhancing antioxidant substances (SOD, GSH), and suppressing oxidative damage markers (MDA, MPO). Further studies indicated that OAG significantly improved intestinal permeability, protected tight junction structures, and restored microvillus morphology. Notably, OAG also played a significant role in maintaining gut microbiota homeostasis. OAG increased the abundance of probiotic bacteria (e.g., Blautia and Ruminococcaceae), reduced the proportion of pathogenic bacteria (e.g., Escherichia-Shigella and *Klebsiella*), and promoted short-chain fatty acid (SCFA) production. This enhanced host intestinal barrier function and maintains homeostasis (Wang C. et al., 2024). Furthermore, multi-omics integration analysis indicated that OAG intervention simultaneously regulated differentially expressed genes, proteins, and metabolites, synergistically influencing multiple pathways including AMPK, HIF-1, MAPK, and PI3K-AKT, ultimately achieving multi-targeted comprehensive intervention against UC. This may thus provide new insights into OAG’s anti-UC effects through anti-inflammatory, antioxidant, and gut microbiota restoration mechanisms.

Anemoside B4 (AB4), a major triterpenoid saponin compound extracted from the roots of the *Pulsatilla chinensis (Bunge) Regel*, has garnered significant attention for its potential efficacy in IBD treatment. Zhang et al. comprehensively validated AB4’s anti-inflammatory activity using a TNBS-induced rat colitis model. The study demonstrated that AB4 (5, 10 mg/kg) significantly alleviated TNBS-induced colonic injury, manifested as slowed weight loss, reduced DAI scores, increased colonic length, and diminished tissue inflammation. Further proteomic analysis revealed that AB4 significantly downregulated S100A9 expression and reduced proinflammatory factors IL-1β, IL-6, and TNF-α by inhibiting the TLR4/MAPK/NF-κB pathway mediated by S100A9. *In vitro* experiments demonstrated that AB4 (25, 100, 400 nM) also inhibited p65, JNK, ERK1/2, and p38 phosphorylation induced by recombinant S100A9 protein, thereby interfering with its downstream proinflammatory signaling (Zhang H. et al., 2021). In summary, AB4 effectively alleviates colonic inflammation by downregulating the S100A9/MAPK/NF-κB signaling axis and inhibiting neutrophil migration and activation, suggesting its potential as a novel therapeutic candidate for UC.

Paeoniflorin (PA), a monoterpene glycoside compound primarily extracted from the dried roots of *Paeonia lactiflora Pall*., exhibits significant anti-inflammatory and anti-apoptotic activities (Zhang and Wei, 2020). In TNBS-induced UC mouse models, PA mitigated inflammatory factor release and inhibited apoptosis by suppressing NF-κB activation. Specifically, PA significantly reduced levels of pro-inflammatory factors (IL-1β, IL-6, IL-12, TNF-α) in colonic tissue while upregulating the anti-inflammatory factor IL-10, thereby alleviating tissue inflammation. Concurrently, PA inhibited the phosphorylation levels of p38 and ERK in MAPK, downregulated the expression of pro-apoptotic proteins Bax and caspase-3/9, and upregulated Bcl-2 expression (Gu et al., 2017). These studies indicate that PA exerts significant anti-inflammatory and anti-apoptotic effects by regulating MAPK/NF-κB axis and modulating the Bcl-2/Bax ratio, demonstrating potential pharmacological value for treating UC.

Pedunculoside (PE) is a triterpenoid saponin extracted from the bark of *Ilex rotunda Thunb.* Previous studies have reported that PE exhibits multiple biological activities, including anti-inflammatory, antitumor, antiviral, lipid-regulating, and antihypertensive effects (Fan et al., 2023). Liu et al. (2020) established DSS-induced UC mouse models to evaluate PE’s protective effects and potential mechanisms in this setting. Experimental results demonstrated that PE treatment (5, 15, 30 mg/kg) significantly improved DSS-induced colon shortening and elevated clinical scores in mice, while reducing pathological damage in colonic tissue, including crypt structure disruption, loss of goblet cells, and inflammatory cell infiltration. In RAW264.7 mouse macrophages, PE (25, 50, 100 μmol/mL) dose-dependently inhibited the mRNA and protein expression of LPS-induced inflammatory mediators IL-1β, IL-6, TNF-α, COX-2, and iNOS within non-cytotoxic concentration ranges. Furthermore, PE suppressed LPS-induced phosphorylation levels of AKT, ERK1/2, JNK1/2, p38, and NF-κB p65 proteins, blocked p65 nuclear translocation, and thereby inhibited activation of the AKT/NF-κB and MAPK inflammatory signaling pathways. In summary, PE alleviates DSS-induced colonic inflammation and tissue damage in mice by downregulating the activation of AKT/NF-κB and MAPK, thereby inhibiting proinflammatory cytokine production. This study suggests that PE may serve as a novel natural product for adjunctive therapy in UC. This study has certain limitations, such as the lack of in-depth investigation into the long-term toxicity, safety margin, *in vivo* metabolic mechanisms, and specific target sites of PE, as well as the absence of clinical research data to support its advancement as a drug candidate.

Asiaticoside (AS), a triterpenoid saponin extracted from *Centella asiatica (L.) Urb.*, has been demonstrated to possess multiple pharmacological properties including anti-inflammatory, anti-fibrotic, and wound-healing effects (Bandopadhyay et al., 2023). Liu et al. found that AS (20, 40, 80 mg/kg) not only improved weight loss, colon shortening, and elevated DAI in DSS-induced colitis mice but also reduced pro-inflammatory factors IL-1β, IL-6, TNF-α, and inflammation-related enzymes MPO, COX-2, and iNOS in colonic tissue, thereby alleviating intestinal inflammatory responses. Concurrently, AS increased expression of intestinal tight junction proteins (claudin-3, occludin, ZO-1), reduced intestinal permeability, protected intestinal barrier integrity, and consequently suppressed damage to intestinal tissue caused by ectopic inflammation. Furthermore, AS modulated gut microbiota composition by enhancing diversity, increasing the relative abundance of beneficial bacteria such as *Lactobacillus* and Prevotella, and reducing harmful bacteria like Oscillospira and *Bacteroides*. This balanced gut microbiome further alleviated colitis symptoms (Liu et al., 2023). These multi-targeted, multi-mechanistic effects indicate AS holds potential clinical application prospects for preventing and treating inflammatory bowel diseases, particularly UC. However, this study has limitations, including its short-term intervention design, which did not explore AS’s long-term efficacy in maintaining remission (e.g., recurrence after discontinuation) or chronic toxicity (e.g., effects of prolonged gastric administration on liver/kidney function and gut microbiota homeostasis). UC requires long-term treatment in clinical practice. Short-term experiments cannot determine AS’s suitability for prolonged use nor rule out potential side effects from long-term administration.

α-Bisabolol is a monocyclic sesquiterpenol naturally occurring in *Matricaria chamomilla L.*, exhibiting diverse bioactivities including anti-inflammatory, analgesic, and antibacterial effects (El Mihyaoui et al., 2022). Experimental results by Venkataraman et al. confirm that α-Bisabolol exhibits significant anti-inflammatory effects in DSS-induced colitis mouse models. It markedly suppressed the expression of inflammatory markers IL-6, IL-1β, TNF-α, and IL-17A, reduced MPO activity, and improved histopathological structure, thereby effectively alleviating intestinal inflammation. Furthermore, α-Bisabolol markedly decreased COX-2 and iNOS expression while reducing tissue nitrite accumulation. Regarding its anti-inflammatory mechanism, α-Bisabolol downregulated MAPK and NF-κB while significantly upregulating PPAR-γ expression in colonic tissue, thereby inducing the transcription of anti-inflammatory genes. In RAW264.7 macrophages and HT-29 intestinal epithelial cells, α-Bisabolol significantly activated PPAR-γ promoter activity and suppressed the expression of inflammation-related chemokines, further supporting its regulatory role at the cellular level (Venkataraman et al., 2022). Therefore, modulating OS mediated by the MAPK may represent a potential mechanism for α-Bisabolol in treating UC.

Stevioside is a natural diterpenoid glycoside extracted *from Matricaria chamomilla L*. Modern pharmacology indicates that stevioside possesses potent anti-inflammatory and antioxidant properties (Peteliuk et al., 2021). Alavala et al. validated stevioside’s potential for UC treatment using DSS-induced acute colitis models. After 14 days of treatment, the Stevioside (50–100 mg/kg) group exhibited a significant reduction in DAI, lessened weight loss, and markedly restored colon length compared to the pathological model group. Stevioside also effectively improved the integrity of the colonic epithelial structure, alleviated submucosal inflammation, lymphocyte infiltration, and crypt destruction. Notably, Stevioside exerted its therapeutic effects by significantly suppressing the expression of inflammatory factors TNF-α, IL-6, COX-2, and iNOS. This was achieved through inhibiting p65 nuclear translocation and IκBα phosphorylation in NF-κB, as well as downregulating p38, ERK, and JNK phosphorylation in MAPK (Alavala et al., 2019). Furthermore, stevioside markedly reduced ROS and nitrate levels while upregulating SOD, CAT, GSH, GST, and HO-1 expression, thereby exerting antioxidant and cytoprotective effects. Through multi-targeted, multi-pathway regulation, stevioside provides protective effects against inflammatory responses, OS, and mucosal damage, offering experimental evidence for its further development as a natural anti-inflammatory agent.

### 5.4 Alkaloid products

Alkaloids represent a highly representative class of naturally occurring nitrogen-containing organic products in nature. Within plants, they are particularly concentrated in specific tissues or secretions of organs such as roots, stems, leaves, fruits, and seeds. These products exhibit remarkable and diverse biological activities due to their complex molecular frameworks and functional group structures, including analgesic, anti-inflammatory, antibacterial, antiviral, antitumor, and cardiovascular regulatory effects.

Nuciferine (NCF), an isoquinoline alkaloid extracted from *Nelumbo nucifera Gaertn.* Which has garnered increasing attention for its therapeutic potential in IBD. Kulhari et al. evaluated NCF’s intervention effects on UC and its molecular mechanisms using *in vivo* and *in vitro* models. *In vivo* experiments demonstrated that oral administration of NCF (20, 40 mg/kg) to DSS-induced UC mice significantly improved DAI scores, restored colonic length and tissue architecture, reduced inflammatory cell infiltration and crypt disruption in tissues, while enhancing GSH and SOD activity and restoring ZO-1 and Claudin-1 protein expression. *In vitro* experiments using LPS-stimulated RAW 264.7 mouse macrophages confirmed these findings. NCF (0.31, 0.63, 1.25 μg/mL) concentration-dependently inhibited the expression of inflammatory factors TNF-α, IL-1β, IL-18, and inducible iNOS, significantly reduced ROS and NO production, suppressed the phosphorylation levels of key proteins in the MAPK (p38, ERK, JNK) and NF-κB (p65, IκBα), while downregulating NLRP3 inflammasome-associated proteins Caspase-1, IL-1β, and IL-18 expression (Kulhari et al., 2023). These studies indicate that NCF exerts its anti-inflammatory and barrier-protective effects by synergistically inhibiting MAPK/NF-κB and NLRP3/Caspase-1. Although this study confirmed NCF’s regulation of multiple inflammation-related pathways, its direct targets or initial signal intervention sites remain unclear. Given the potential for cross-regulatory mechanisms among MAPK, NF-κB, and NLRP3, future studies should further clarify which pathway represents the dominant upstream mechanism.

Berberine (BBR) is a natural isoquinoline alkaloid primarily extracted from *Coptis chinensis Franch.* which has garnered significant attention due to its prominent pharmacological properties, including anti-inflammatory, antioxidant, and antitumor effects (Wang et al., 2017). Jia et al. investigated the therapeutic potential of BBR in acetic acid-induced rat UC models. Experimental results demonstrated that seven consecutive days of oral BBR administration (25, 50 mg/kg) significantly improved macroscopic and histological lesions in colonic tissue. It suppressed inflammatory mediators (TNF-α, IL-1β, IL-6, PGE_2_) and MPO activity while enhancing antioxidant defense systems (SOD, CAT, GPx, GR) activity, restoring GSH levels, and reducing MDA and NO content. Furthermore, BBR significantly reduced Bax and caspase-3 levels while increasing Bcl-2 expression, thereby exerting an anti-apoptotic protective effect. These findings indicate that BBR exerts antioxidant and anti-inflammatory effects by downregulating p38 MAPK and iNOS gene expression while upregulating Nrf2 and its downstream target HO-1 gene expression (Jia et al., 2020). This study has limitations. Although high-dose BBR showed significant effects, it did not evaluate its toxicology or long-term safety, lacking assessment of adverse effects. In summary, this study validated that BBR alleviates pathological damage in colitis through synergistic multi-pathway actions (antioxidant, anti-inflammatory, anti-apoptotic) and proposed its potential mechanism involving regulation of Nrf2 and p38 MAPK, providing theoretical support for developing novel plant-derived anti-inflammatory drugs.

### 5.5 Polysaccharide products

Polysaccharides are naturally occurring macromolecular carbohydrates widely distributed in plant organs (roots, stems, etc.), algae, fungi, and animal tissues. Structurally based on a backbone formed by monosaccharide units linked via glycosidic bonds and functional groups such as hydroxyl and carboxyl groups, they exhibit significant anti-inflammatory activity and immunomodulatory effects. As important candidates for natural anti-inflammatory drugs, they offer therapeutic directions for chronic inflammatory diseases.

Mesona chinensis Benth polysaccharides (MBP) are bioactive polysaccharides extracted from *Platostoma palustre (Blume) A.J.Paton*, exhibiting potent anti-inflammatory and gut microbiota-modulating pharmacological activities (Pan et al., 2024). Lu et al. investigated MBP’s effects in DSS-induced UC mouse models and compared its therapeutic efficacy with 5-ASA. The study established groups receiving different MBP doses (100, 200, 300 mg/kg) via continuous oral administration for 14 days. Results showed that DSS significantly induced weight loss, colon shortening, elevated DAI scores, tissue structural disruption, and inflammatory cell infiltration. All MBP dose groups and the 5-ASA group (200 mg/kg/d) significantly improved these symptoms, with the MBP (200 mg/kg) group demonstrating the most optimal effect. Furthermore, MBP reduced levels of proinflammatory factors TNF-α and IL-1β in colonic tissue, increased SOD, GSH-Px, and CAT activity, decreased MDA levels, elevated anti-inflammatory factor IL-10 levels, inhibited MPO activity, and alleviated inflammatory responses. 16S rRNA sequencing revealed that MBP significantly improved gut microbiota imbalance by increasing beneficial bacteria such as *Lactobacillus* and Coprococcus while reducing harmful bacteria like *Helicobacter* and Prevotella, thereby regulating microbial diversity and structure. Its efficacy surpassed that of the 5-ASA group. Mechanistically, MBP reduced inflammation and apoptosis by inhibiting phosphorylation of key proteins in TLR4/MAPK/NF-κB and modulating the Bcl-2/Bax ratio (Lu et al., 2022). Although the 5-ASA group served as a control to evaluate MBP efficacy, this study did not assess the potential for combined use of both agents nor evaluate the potential toxicity of high-dose MBP.

Pyrus pashia Buch polysaccharide (PPBP), primarily extracted from *Pyrus ussuriensis Maxim.*, exhibits anti-inflammatory/antioxidant pharmacological properties (Om et al., 2022). This study evaluated PPBP’s mechanisms in improving intestinal inflammation, regulating gut microbiota, and enhancing SCFA production using DSS-induced colitis mouse models. Results demonstrated that both SASP (200 mg/kg) and PPBP (70, 320 mg/kg) alleviated symptoms including weight loss, colon shortening, and elevated inflammatory markers in mice. Concurrently, they reduced OS levels in colonic tissue by downregulating pro-inflammatory factors (TNF-α, IL-6, IL-1β, iNOS) and antioxidants (SOD, GSH-PX), thereby mitigating intestinal mucosal damage (Zhang et al., 2025a). At the molecular level, PPBP inhibited the activation of MAPK/P38 and NF-κB P65, reduced IκBα phosphorylation, and blocked pro-inflammatory signal transduction, consequently decreasing the release of inflammatory mediators. Concurrently, PPBP regulated SCFAs-ERK-MSK to maintain stable ERK/MSK, effectively promoting short-chain fatty acid synthesis (particularly acetate and butyrate), thereby supporting intestinal barrier integrity restoration. This indicated that PPBP’s role in alleviating OS and inflammation positively influences the inhibition of UC progression.

Zingiber officinale polysaccharide (ZOP), a natural polysaccharide extracted from *Zingiber officinale Roscoe*, exhibits diverse pharmacological activities with significant anti-inflammatory, antioxidant, and gut-protective effects (Mao et al., 2019). Experiments by Jing et al. demonstrated that ZOP-1 (500, 1,000 mg/kg) improved clinical symptoms in rats, including weight loss, colon shortening, and mucosal damage. It reduced the expression of pro-inflammatory factors such as TNF-α, IL-6, and IL-1β, while upregulating SOD expression and suppressing MDA and MPO levels. Concurrently, ZOP-1 repaired colonic epithelial barrier function by upregulating Occludin and ZO-1 protein expression. It enhanced gut microbiota diversity by increasing the abundance of probiotics like Muribaculaceae and reducing the proportion of pathogens such as Proteobacteria, thereby regulating intestinal immunity and barrier homeostasis. Moreover, ZOP-1’s significant suppression of key protein expression levels in the TLR4/MyD88/NF-κB and MAPK indicated its protective role through immune-inflammatory signaling intervention. Its comprehensive effects at high doses rivaled those of SASP (9 mg/kg) (Jing H. et al., 2025). Although this study highlighted the efficacy of ZOP-1 in treating UC to some extent, the experiments were limited to mice and did not include higher-level assessments such as organoid models, humanized models, or even preclinical pharmacokinetic studies, limiting its clinical extrapolation.

Polysaccharides of C. nudiflora Hook (CNLP) which extracted from *Callicarpa nudiflora Hook. and Arn.*, have garnered significant attention due to their anti-inflammatory, antioxidant, and immunomodulatory activities. Feng et al. established DSS-induced colitis mouse models and found that CNLP (100, 200, 400 mg/kg) significantly reduced DAI, MPO, MDA, and the expression of proinflammatory factors TNF-α, IL-6, and IL-1β in a dose-dependent manner, with SASP (10 mg/kg) showing similar effects. Concurrently, CNLP restored the expression of tight junction proteins such as ZO-1, Occludin, Claudin-1, and MUC-2, thereby enhancing mucosal barrier function. The anti-inflammatory effects of CNLP were achieved through multi-pathway synergism: on one hand, it inhibited the phosphorylation of p38 MAPK and JNK while blocking the degradation of inhibitory IκBα and the nuclear translocation of NF-κB p65, suppressing inflammatory responses at the signaling pathway level; On the other hand, CNLP significantly downregulated the transcription of inflammatory-related factors such as TLR4, COX-2, and iNOS, further enhancing anti-inflammatory effects at the molecular level. Additionally, CNLP improved DSS-induced gut dysbiosis by increasing the abundance of probiotics like *Lactobacillus* johnsonii and promoting the production of SCFAs such as acetate, propionate, and butyrate, thereby optimizing the intestinal microenvironment (Feng et al., 2024). However, this study did not address safety considerations or clinical translation pathways. For potential development as a functional food or drug candidate, toxicological data and long-term safety evaluations are lacking and require supplementation in future research.

### 5.6 Coumarin products

Coumarins are natural phenylpropanoid derivatives widely distributed in plant tissues of the Apiaceae and Rutaceae. Based on the benzo-α-pyrone core structure with functional groups like hydroxyl and methoxy, they exhibit significant anti-inflammatory, antioxidant, and mucosal protective activities. As important candidates for natural anti-inflammatory drugs, they offer new therapeutic strategies for diseases like UC.

7-hydroxycoumarin (7-HC) is a natural coumarin compound widely distributed in *Phaseolus vulgaris L.* In recent years, it has garnered extensive attention for its diverse biological activities, including antitumor, anti-inflammatory, and antioxidant effects (Wu et al., 2020). This study confirmed the therapeutic efficacy of 7-HC in DSS-induced UC mouse models through *in vivo* experiments. Liu et al. found that 7-HC (25, 50, 100 mg/kg) significantly improved colonic tissue damage in a dose-dependent manner, reduced levels of inflammatory cytokines TNF-α and IL-1β, enhanced antioxidant enzyme SOD activity, and suppressed MPO expression, thereby alleviating OS (Liu K. et al., 2024). Furthermore, experiments confirmed that 7-HC exerts its anti-inflammatory effects by inhibiting downstream inflammatory responses through suppression of ERK, JNK, p38, and their phosphorylation levels in MAPK. Notably, this study also revealed that 7-HC effectively corrects DSS-induced dysbiosis by regulating the structure and diversity of the gut microbiota (Liu M. et al., 2024). However, this study has limitations, such as mechanism validation remaining focused on *in vivo* experiments, with insufficient preclinical pharmacokinetic and toxicological research. Although 7-HC demonstrates good biocompatibility, its long-term safety, metabolic characteristics, and *in vivo* exposure levels remain unexplored.

Pimpinellin is a major coumarin compound extracted from *Zanthoxylum asiaticum (L.) Appelhans, Groppo and J. Wen*. Modern pharmacology indicates that pimpinellin possesses anti-inflammatory and antioxidant bioactivities (Yang et al., 2024). In this experiment, mice were modeled with 2% DSS for 7 days and concurrently treated with pimpinellin (50, 75, 100 mg/kg) via oral gavage for 21 days. Results demonstrated that pimpinellin ameliorated DSS-induced phenotypes including body weight loss, colon shortening, and elevated DAI scores. It also mitigated histopathological damage in colonic mucosa, enhanced expression of ZO-1, occludin, and claudin-3, and suppressed elevated proinflammatory factors (TNF-α, IL-1β, IL-6) and excessive overexpression of COX-2/iNOS proteins. Furthermore, pimpinellin downregulated p-p38, p-JNK, p-ERK, p-p65, and p-IκB expression while increasing the abundance of beneficial bacteria such as Lactobacillaceae and S24-7 and reducing pathogenic bacteria like Enterobacteriaceae and *Shigella*. FMT experiments further confirmed that pimpinellin-treated fecal microbiota alleviated UC symptoms, validating its microbiota-mediated effects (Lv et al., 2025). While this study demonstrates pimpinellin’s anti-inflammatory efficacy, comparative studies or combination therapy with first-line drugs like 5-ASA or sulfasalazine would provide greater practical guidance.

Osthole is a natural coumarin compound isolated from the fruits of *Cnidium monnieri (L.) Cusson*, exhibiting anti-inflammatory, antibacterial, and immunomodulatory pharmacological properties. This study elucidated Osthole’s anti-inflammatory potential through *in vivo* and *in vitro* experiments and further explored its mechanisms. *In vivo* experiments demonstrated that Osthole (10, 20, 40 mg/kg) alleviated weight loss, improved DAI, delayed colon shortening, and reduced histological damage in UC mice, with therapeutic effects comparable to Mesalazine (200 mg/kg). *In vitro*, Osthole (26, 50, 100 μM) significantly inhibited the release of NO, PGE_2_, TNF-α, and IL-6 in LPS-induced RAW 264.7 macrophages. This therapeutic mechanism may be associated with the suppression of p38 MAPK protein phosphorylation levels (Fan et al., 2019). In this study, although Osthole is hypothesized to act on MAPK/p38, its direct molecular targets remain unidentified. There is a lack of binding site or target validation experiments, such as molecular docking or target enrichment analysis.

### 5.7 Other products

Shikimic acid (SA) is a monomeric compound extracted from the Chinese medicinal herb *Illicium verum Hook. f.* It exhibits anti-inflammatory and analgesic effects (Sheng et al., 2023). Li et al. systematically evaluated SA’s therapeutic potential using DSS-induced UC mouse models. The study demonstrated that SA (10–50 mg/kg) significantly alleviated weight loss, reduced DAI, improved colonic pathological damage, and enhanced intestinal barrier function without observable toxicity. SA inhibited the phosphorylation of key proteins in NF-κB/MAPK, decreased TNF-α, IL-1β, and IFN-γ expression, and upregulated IgG levels to exert anti-inflammatory effects (Li X. et al., 2023). Furthermore, SA modulated gut microbiota composition by increasing Bacteroidetes and decreasing Proteobacteria abundance, contributing to improved intestinal microecology. Although this study revealed SA’s potential in multi-targeted anti-inflammation, its clinical application requires further investigation into dose optimization, long-term safety, and precise regulation of its mechanisms of action.

N-Acetyldopamine Dimer (NADD) is a small-molecule compound isolated from *Isaria cicadae Miquel*, exhibiting antioxidant, anti-aging, anti-inflammatory, and antitumor effects (Thapa et al., 2025). Huang et al. established UC mouse models induced by 2.5% DSS and administered varying doses of NADD via continuous gavage to evaluate its therapeutic potential. Treatment with NADD (5, 10, 20 mg/kg) significantly ameliorated DSS-induced symptoms including weight loss, colon shortening, tissue damage, and elevated inflammatory markers. Histologically, it restored goblet cell numbers, mucus layer integrity, and crypt architecture. Mechanistically, NADD dose-dependently downregulated mRNA and protein levels of proinflammatory factors including TNF-α, IL-6, IL-1β, iNOS, and NF-κB in DSS-treated colon tissue. Further *in vitro* experiments in RAW264.7 macrophages demonstrated that NADD reversed LPS-induced cellular morphological alterations and proinflammatory factor expression while significantly inhibiting NO production without affecting cellular viability. Transcriptome sequencing and KEGG enrichment analysis further revealed that NADD significantly suppressed LPS-upregulated gene expression. The differentially expressed genes were predominantly enriched in the NF-κB and MAPK inflammatory pathways. NADD concentration-dependently inhibited LPS-induced phosphorylation of IKK, p65, and IκBα, while downregulating phosphorylation levels of JNK, ERK, and p38 (Huang et al., 2022). The above studies indicate that NADD significantly alleviates DSS-induced UC pathological symptoms and exerts its anti-inflammatory effects by inhibiting the activation of NF-κB and MAPK. However, this experiment still has some limitations. For example, although RNA-seq was performed and relevant differentially expressed genes (DEGs) and pathway enrichment were reported, no further functional validation (such as knockdown/overexpression experiments) was conducted for key candidate genes.

Amauroderma rugosum (AR) is a medicinal fungus with anti-inflammatory and antioxidant potential, primarily isolated from basidiomycetes of the *Ganodermataceae*. It is commonly used to treat various conditions including inflammation, gastrointestinal disorders, and cancer (Zheng et al., 2022). Li et al. demonstrated that oral administration of AR extract (50, 100, 200 mg/kg) in DSS-induced UC mouse models restored intestinal immune homeostasis by inhibiting M1 macrophage polarization and promoting M2 macrophage polarization. This resulted in increased colon length, improved body weight, reduced DAI, and decreased spleen index. Hematoxylin and eosin (HE) staining revealed that AR effectively alleviated mucosal edema, adhesion, and epithelial cell destruction in colonic tissue. These phenotypic changes correlated with downregulated MAPK activity, reduced release of proinflammatory mediators (NO, TNF-α, IL-1β), and increased expression of anti-inflammatory markers (CD206, Arg-1). *In vitro* experiments further validated AR’s ability to inhibit phosphorylation in MAPK-related pathways, thereby suppressing inflammatory signaling (Li J. et al., 2024). Although this study demonstrated the potential therapeutic effect of AR extract in alleviating DSS-induced UC, the absence of a positive control group in the experimental design precludes an objective assessment of the efficacy difference between AR and existing standard treatments. It also makes it difficult to determine whether the observed effect strength possesses clinical translational feasibility.

Extract of Atractylodis Rhizoma (EEAR) demonstrated significant preventive effects against DSS-induced acute UC, which isolated from *Atractylodes lancea (Thunb.) DC*. Xiong et al. observed in UC mouse models that EEAR (555, 1,110 mg/kg) significantly improved weight loss, colon shortening, elevated spleen index, goblet cell loss, histopathological damage, and increased MPO activity. It also markedly reduced inflammatory markers TNF-α, IL-6, IL-1β. These effects were comparable to those of SASP (250 mg/kg). Mechanistic studies revealed that EEAR maintained intestinal barrier integrity by upregulating ZO-1 and Occludin proteins while inhibiting phosphorylation levels in MAPK (p-JNK, p-ERK, p-p38) and NF-κB (p-p65, p-IκBα), thereby suppressing inflammatory responses. *In vitro* experiments further demonstrated that EEAR (at concentrations of 12.5 and 25 μg/mL) significantly reduced inflammatory cytokine levels and NO production in LPS-stimulated RAW264.7 cells, validating the *in vivo* findings (Lin et al., 2022). This study effectively elucidates how EEAR exerts anti-UC effects by modulating MAPK/NF-κB and maintaining intestinal barrier function, providing crucial theoretical support for developing natural medicines based on Atractylodes macrocephala. However, certain limitations exist: while *in vivo* experiments validated the effective dose range, they did not further assess pharmacokinetics, tissue distribution, or toxicity accumulation risks, limiting feasibility analysis for clinical translation.

Citrus unshiu peel water extract (CUP), derived from the dried peel of *Citrus aurantium f. deliciosa (Ten.) M. Hiroe*, has demonstrated pharmacological properties including anti-inflammatory and antioxidant effects (Kim and Lim, 2020). Lee et al. Established acute UC mouse models using 5% DSS and validated CUP’s therapeutic efficacy. *In vivo* results showed that both CUP (100, 200 mg/kg) and SASP (100 mg/kg) significantly improved DSS-induced weight loss, colon shortening, inflammatory cell infiltration, and histological damage. They also markedly reduced serum ROS levels and colonic tissue MDA and MPO content. Mechanistic studies revealed that CUP downregulated phosphorylation of MAPK (p38, ERK, JNK) and transcription factors c-Fos and c-Jun, while inhibiting IκBα phosphorylation and NF-κB p65 nuclear translocation. This reduced expression of inflammatory mediators including iNOS, COX-2, TNF-α, IL-6, and IL-1β (Lee et al., 2022). In summary, CUP may exert antioxidant and anti-inflammatory effects by regulating PI3K/Akt and its downstream signaling axes (MAPK, NF-κB), thereby producing significant protective effects against UC. Notably, although this study included low-and high-dose groups, a systematic analysis of the dose-response relationship was not conducted, and some indicators did not show clear dose dependency. The underlying mechanisms warrant further investigation in subsequent studies.

Anneslea fragrans extract (AFE) derived from *Anneslea fragrans Wall.*, which has demonstrated anti-inflammatory and antioxidant pharmacological effects. Deng et al. experimentally highlighted that AFE (200–600 mg/kg) suppressed the activation of NF-κB and MAPK in DSS-induced mouse colon tissue, reduced p65 and IκBα phosphorylation levels, and significantly upregulated the expression of intestinal tight junction proteins ZO-1, Occludin, and Claudin-1 in DSS-induced mouse colon tissue, thereby enhancing intestinal barrier integrity and alleviating inflammation. Its therapeutic effect was comparable to that of SASP (450 mg/kg). This effect was accompanied by a marked decrease in proinflammatory factors TNF-α, IL-1β, and IL-6 in the inflamed mouse colon and serum, along with upregulation of anti-inflammatory factors IL-4 and IL-10. Consequently, MPO activity and OS levels in tissues were reduced, while SOD and GSH activity increased, ultimately alleviating UC pathological damage, decreasing DAI, and restoring colon length (Deng et al., 2021). Although the present study demonstrates the promising efficacy of AFE extract in the UC model, the lack of research on its bioavailability, pharmacokinetic parameters, and long-term toxicity limits its feasibility as a clinical candidate drug.

Flower extract of Caragana sinica (FEC) has been reported to possess significant anti-inflammatory and antioxidant activities, found in *Caragana sinica (Buc’hoz) Rehder*. Li et al. demonstrated that oral administration of FEC (250, 500 mg/kg) improved symptoms in DSS-induced UC mice while reducing colonic lesion severity and delaying colonic shortening. This effect primarily occurs through inhibition of the TLR4/NF-κB and TLR4/MAPK, manifested by downregulating proinflammatory factors TNF-α, IL-1β, IL-6, and key signaling proteins p-NF-κB p65, p-p38, p-ERK, while upregulating anti-inflammatory factor IL-10 and antioxidant proteins SOD, CAT, and GSH (Li et al., 2021). Despite FEC’s promising therapeutic potential, its clinical application faces challenges due to its complex chemical composition and unclear water solubility and bioavailability data. Future studies may enhance the solubility and bioavailability of active components through novel delivery systems such as nanocarriers or liposomes.

Syringaresinol (SYR), a natural lignan compound found in roots, stems, leaves, fruits, or seeds of various plants such as *Dracaena draco (L.) L.*, exhibits significant antioxidant and anti-inflammatory properties, demonstrating potential pharmacological effects in UC treatment (Wang Y. et al., 2023). Liu et al. evaluated SYR’s effects on intestinal epithelial barrier function and inflammatory responses using LPS-induced Caco-2 cell models and DSS-induced UC mouse models. *In vitro* experiments revealed that SYR (25, 50, 100 μM) significantly increased the trans-epithelial electrical resistance (TEER) values of Caco-2 cells, restored the expression and localization of tight junction proteins (e.g., ZO-1, occludin, claudin-1, E-cadherin), while significantly downregulating the expression levels of inflammatory mediators TNF-α, IL-6, IFN-γ, and COX-2, demonstrating a dose-dependent anti-inflammatory effect. *In vivo* experiments demonstrated that SYR (10, 20, 40 mg/kg) significantly alleviated DSS-induced UC symptoms in mice, including improvements in weight loss, colon shortening, elevated spleen coefficient, and intestinal structural integrity. Its mechanism primarily involves inhibiting the phosphorylation of PI3K, Akt, NF-κB, MAPK, and GSK-3β (Liu Y. et al., 2024). Collectively, these findings indicate that SYR holds broad clinical potential for UC management by restoring the intestinal epithelial barrier and regulating inflammatory responses and their associated signaling pathways. This study has limitations, such as establishing only an acute DSS-induced UC model without covering chronic or recurrent UC models, which may restrict its applicability during clinical translation.

Sinigrin is a naturally occurring aliphatic glucosinolate found in cruciferous vegetables such as *Brassica oleracea L*. It has garnered significant attention for its anti-inflammatory, antioxidant, and antitumor properties. Kotipalli et al. found that Sinigrin (15, 30 mg/kg) effectively alleviated DSS-induced UC in mice, manifested as reduced weight loss, shortened colon length, alleviated splenomegaly, and decreased DAI. Additionally, Sinigrin significantly suppressed the expression of pro-inflammatory cytokines TNF-α, IL-6, IL-1β, and IL-17A, restored antioxidant enzyme levels (GSH, SOD, CAT), and reduced OS markers (MDA, NO). Mechanistically, Sinigrin achieves its protective effect on the intestinal barrier by inhibiting phosphorylation in MAPK (including p38, JNK, ERK, and downstream cJUN), thereby reducing the inflammatory cascade (Kotipalli et al., 2023). These studies suggest focusing on Sinigrin for regulating OS and gut microbiota, offering a novel approach to intervene in UC through this regulatory process.

Phillygenin (PHI) is a natural bioactive compound isolated from the dried fruits of *Forsythia suspensa (Thunb.) Vahl*, a plant belonging to the Oleaceae family which exhibits anti-inflammatory, antioxidant, and hepatoprotective activities. Xue et al. conducted *in vivo* experiments using 3% DSS-induced UC mouse models, administering PHI (25, 50, 100 mg/kg) for 8 days, with Mesalazine (100 mg/kg) as the positive control. Results showed that PHI significantly alleviated weight loss, colon shortening, pathological damage, and hepatosplenomegaly in DSS-treated mice while improving intestinal mucosal barrier function, with therapeutic effects comparable to Mesalazine. PHI also reduced MDA levels, inflammatory mediators TNF-α, IL-1β, IL-6, and MPO activity in intestinal tissues, while upregulating ZO-1, E-cadherin, and occludin expression and restoring SOD and IL-10 levels. Mechanistic studies indicate that PHI promotes TLR4 and its downstream Src inhibition, reducing activation of MAPK (p38, JNK) and NF-κB. Furthermore, *in vitro* experiments similarly demonstrate that PHI exerts anti-inflammatory effects via MAPK (Xue et al., 2023). This study provides robust *in vitro* and *in vivo* evidence supporting PHI for UC treatment. However, the study’s short duration limited evaluation of long-term efficacy and drug toxicity, constraining clinical translation.

Arbutin, a natural antioxidant primarily found in *Arctostaphylos uva-ursi (L.) Spreng.*, exhibits pharmacological effects including anti-inflammatory, antioxidant, and antitumor properties (Nahar et al., 2022). Zhang et al. reported that Arbutin (50, 100 mg/kg) significantly improved weight loss, colon shortening, and pathological damage in UC mice. It also suppressed the expression of inflammatory factors TNF-α and IL-6, enhanced the expression of ZO-1, Occludin, and Claudin-1, and upregulated the anti-inflammatory factor IL-10. Mechanistically, arbutin inhibited the phosphorylation levels of p-JNK, p-p38, and p-ELK1, suggesting its action may be mediated through MAPK/ELK1 (Zhang C. et al., 2021). These findings demonstrate that arbutin exerts significant protective effects in DSS-induced UC models by modulating inflammation, apoptosis, and intestinal barrier integrity, suggesting potential therapeutic value. Although this study revealed alterations in key nodes of MAPK/ELK1 and validated its regulatory role in inflammation and apoptosis, theoretically supporting Arbutin’s intervention mechanism, the absence of corresponding inhibitor interventions or knockout experiments precludes direct confirmation of causal relationships within the pathway, leaving the mechanism validation incomplete.

Atractylodin (ALT) is a natural compound isolated from the dried rhizome of *Atractylodes macrocephala Koidz.*, a plant belonging to the Asteraceae family. Existing evidence indicates that ALT possesses pharmacological properties including anti-inflammatory, immunomodulatory, and antitumor effects (Heo et al., 2023). Research by Qu et al. revealed that ALT (10–20 mg/kg) alleviated weight loss, colon shortening, and tissue inflammatory damage in DSS-induced colitis mice. It also restored expression of intestinal epithelial barrier proteins ZO-1, occludin, and MUC2, maintaining mucus layer integrity, with efficacy comparable to the positive control drug SASP (250 mg/kg). Furthermore, ALT inhibited MAPK activation in macrophages, reduced secretion of proinflammatory factors TNF-α, IL-6, and IL-1β, suppressed iNOS expression, and downregulated TNF-α translation by decreasing GAPDH activity and malondialdehyde levels. 16S rRNA sequencing results further revealed that ALT significantly increased the abundance of gut probiotics Akkermansia and Alistipes while reducing pathogenic bacteria *Helicobacter* and Desulfovibrio, promoting gut microbiota remodeling (Qu et al., 2021). Collectively, ALT may exert protective effects by synergistically regulating immune inflammatory responses and barrier function through the “gut microbiota–mucosal barrier–macrophage metabolism” axis.

Alliin, a naturally occurring organosulfur phytochemical derived from *Allium sativum L*., has demonstrated anti-inflammatory and antibacterial bioactivities (Liu M. et al., 2022). *In vivo* experiments showed that alliin (500 mg/kg) effectively alleviated clinical colitis phenotypes in UC mice, including weight loss, intestinal hemorrhage, and diarrhea, while significantly improving histopathological damage and reducing colonic MPO, MDA, and NO levels. *In vitro* experiments demonstrated that Alliin treatment in RAW264.7 cells dose-dependently inhibited LPS-induced expression of inflammatory factors TNF-α, IL-1β, IL-6, and iNOS, while reducing NO production. Further mechanistic studies revealed that Alliin inhibited phosphorylation of p38, JNK, and ERK1/2 in MAPK, thereby blocking nuclear translocation of NF-κB, AP-1, and STAT-1 to suppress downstream inflammatory factor expression (Shi et al., 2017). In summary, alliin exerts a multi-target synergistic effect in alleviating intestinal inflammation by regulating inflammatory signaling pathways such as MAPK/NF-κB/AP-1/STAT-1, demonstrating its potential as a functional natural product in anti-inflammatory therapy.

Octacosanol is a high-content saturated fatty alcohol present as a wax ester in natural plants such as *Saccharum sinense Roxb.*, demonstrated to possess anti-inflammatory and antioxidant pharmacological effects (Miao et al., 2022). *In vivo* experiments involved Guo et al. administering octacosanol (100 mg/kg) via gavage to UC mice for 13 consecutive days. Results indicated that octacosanol ameliorated DSS-induced weight loss, diarrhea, and other symptoms while reducing histological damage in colonic tissue. Compared to the model group, octacosanol treatment significantly suppressed mRNA and protein expression of inflammatory mediators TNF-α, IL-1β, IL-6, and iNOS in colonic tissue, concurrently downregulating MPO, MDA, and MAPK levels. *In vitro* studies further demonstrated that octacosanol effectively suppressed inflammatory factor expression in LPS-stimulated RAW264.7 cells, inhibited nuclear translocation of p65 and c-Jun, reduced transcriptional activity of NF-κB and AP-1, and downregulated phosphorylation of p38, JNK, and ERK in MAPK, thereby indirectly affecting the activation of these transcription factors (Guo et al., 2017). Collectively, these findings suggest that octacosanol modulates inflammatory responses by blocking MAPK/NF-κB/AP-1 to downregulate proinflammatory factor expression. However, this study did not further investigate its upstream receptors or downstream target gene networks, limiting comprehensive understanding of its mechanism of action.

Chebulagic acid (CA) is a hydrolyzable tannin-type natural product isolated from the dried ripe fruits of *Terminalia chebula Retz.*, which exhibits pharmacological properties including antitumor, anti-inflammatory, and antioxidant effects (Zhao et al., 2023). Zhang et al. demonstrated CA’s efficacy in alleviating DSS-induced acute UC and elucidated its potential mechanisms. CA (20–100 mg/kg) improved weight loss, DAI scores, colon shortening, and histological damage scores in colitis-affected mice. It elevated SOD, GSH-PX, ZO-1, and Occludin levels while reducing MDA content and suppressing MAPK expression. Furthermore, CA suppressed the growth of harmful bacteria (e.g., *Escherichia*_*Shigella* and *Clostridium*_sensu_stricto_1) while promoting the proliferation of probiotics (e.g., Faecalibacterium, Dubosiella, and Muribaculaceae), thereby improving dysbiosis (Zhang et al., 2025b). In summary, CA protects the colonic mucosa by exerting antioxidant, anti-inflammatory, mucosal barrier-enhancing, and gut microbiota-modulating effects through MAPK. However, while this study confirmed MAPK involvement in CA’s actions, it did not further elucidate key targets or whether other signaling pathways co-regulate these effects, lacking direct validation of molecular targets.

## 6 Discussion

UC is a chronic inflammatory disease affecting the digestive system, primarily characterized by recurrent inflammation of the colon and rectum, leading to severe symptoms such as diarrhea, abdominal pain, and bloody stools. The pathological mechanisms of this disease remain incompletely understood, but research indicates that immune dysregulation, gut microbiota imbalance, and dysregulated activation of multiple inflammatory signaling pathways are key factors in its onset and progression. As a major intracellular signaling pathway, MAPK is involved in UC pathogenesis. Its abnormal activation leads to excessive immune cell activation and dysregulated release of inflammatory mediators, thereby exacerbating chronic intestinal inflammation. Consequently, targeting MAPK has become a key therapeutic strategy for UC. Natural products, characterized by their wide availability, structural diversity, low toxicity, and multi-targeted action, represent promising candidates for UC treatment. Various natural products, including flavonoids, phenolics, and terpenoids, exert anti-inflammatory, antioxidant, and intestinal barrier repair effects by modulating the phosphorylation levels of key MAPK molecules. These products effectively alleviate UC pathological symptoms. Compared to conventional chemotherapeutic agents, natural products offer unique therapeutic advantages through multiple synergistic mechanisms such as regulating the gut microbiota while exhibiting reduced toxicity and side effects.

MAPK does not operate in isolation but functions as a pivotal molecular hub, forming a dynamic network of cross-regulatory interactions with pathways such as NF-κB, Nrf2, PI3K/Akt, and NLRP3, collectively contributing to the pathological process of UC. Specifically, a significant pro-inflammatory synergistic effect exists between MAPK and NF-κB: Activation of MAPK family members not only directly regulates the expression of downstream inflammation-related transcription factors but also promotes IκB degradation, accelerating NF-κB nuclear translocation and further amplifying pro-inflammatory signals. Simultaneously, sustained MAPK overactivation suppresses Nrf2-mediated antioxidant defense mechanisms, disrupting the intestinal mucosal oxidation/antioxidation equilibrium and exacerbating apoptosis and mucosal injury. Furthermore, PI3K/Akt can amplify MAPK as an upstream or parallel regulatory pathway, affecting intestinal cell survival. MAPK participates in the “priming” process of NLRP3 inflammasomes, promoting the maturation and release of inflammatory mediators like IL-1β, inducing epithelial cell pyroptosis, and further exacerbating intestinal inflammation. Therefore, targeting the regulation of MAPK and its cross-linking nodes with other pathways holds promise for synergistically improving intestinal immune imbalance and oxidative damage, offering a new direction for precision treatment of UC.

Combination therapy is a key strategy in current UC clinical management. The co-administration of natural products with first-line drugs provides a novel approach to enhance efficacy and reduce adverse effects. Despite being foundational to UC management, standard therapies (aminosalicylates, immunosuppressants, biologics) are often limited as monotherapy. Challenges include efficacy plateaus, secondary resistance, and serious adverse effects from prolonged high-dose use, such as opportunistic infections, osteoporosis, and hepatorenal toxicity. Against this backdrop, natural products with their unique pharmacological properties, hold promise as “sensitizers” or “adjuvants” in clinical interventions. Mechanistically, abnormal activation of the MAPK serves as a key compensatory survival signal enabling cells to evade conventional drug-induced killing and develop resistance. Natural products can specifically inhibit the MAPK cascade, blocking this compensatory pathway and thereby restoring or enhancing the body’s sensitivity to conventional drugs. More importantly, this combination strategy exhibits a significant “dose-sparing effect”. It allows for substantial reduction in the dosage of first-line drugs with stronger toxic side effects while maintaining or even enhancing anti-inflammatory efficacy. This improves long-term patient prognosis and treatment compliance. Additionally, many natural products possess both excellent antioxidant and mucosal repair functions. They can counteract oxidative stress damage induced during the metabolism of chemotherapeutic agents, providing an additional protective barrier for the damaged intestinal epithelium.

Although natural products targeting MAPK show broad potential in basic research for treating UC, current studies face numerous limitations that severely hinder their translation from laboratory to clinical settings. First, there is a significant disconnect between experimental models and clinical pathological features: Most existing studies rely on DSS- or TNBS-induced acute UC mouse models. While these rapidly replicate intestinal inflammatory phenotypes, they struggle to mimic core clinical features of UC, such as chronic recurrence, progressive mucosal damage, and immune microenvironment remodeling. The inherent limitations of a single animal model lie in its inability to account for the individual heterogeneity among UC patients, including variations in disease stage, inflammatory involvement, and genetic background, which in turn constrains the translational value of the research. Second, evaluation systems lack standardization: Significant variations exist in efficacy assessment metrics across studies. Some focus solely on macroscopic phenotypes like weight changes and diarrhea scores, while few incorporate intestinal mucosal histology grading or inflammatory cytokine detection which all lacking unified quantitative standards. Concurrently, studies on the molecular mechanisms by which natural products regulate the MAPK largely remain at the level of key kinase phosphorylation, failing to delve into the molecular networks of cross-regulatory interactions between pathways. Additionally, investigations into synergistic mechanisms such as intestinal barrier repair and gut microbiota regulation remain insufficiently systematic, resulting in limited comparability and persuasiveness of research conclusions. Third, safety evaluation and quality control are inadequate: Most studies focus on anti-inflammatory efficacy while neglecting long-term safety assessment, failing to conduct systematic investigations into chronic toxicity, hepatic/renal function impacts, or reproductive toxicity. Given the complex composition of natural products, where active ingredient content varies due to factors like origin, harvest season, and extraction methods, existing research lacks unified quality control standards and insufficiently evaluates interactions with first-line drugs, increasing potential risks for clinical application. Finally, translational research remains weak: Current studies are predominantly confined to *in vitro* cell experiments or animal models, lacking support from large-scale, multicenter clinical trials.

To address these limitations, future research should advance in the following areas to accelerate the clinical translation of natural products targeting MAPK for UC treatment. First, establish more clinically relevant UC models: Develop chronic recurrent UC models integrated with humanized intestinal organoid or humanized mouse models to precisely mimic the pathological features and immune microenvironment of clinical UC patients, enhancing the clinical relevance of research outcomes. Simultaneously, establish a patient-stratified model system to screen for specific natural products targeting the pathological characteristics of different UC subtypes. Second, establish standardized evaluation systems and deepen mechanism research: Formulate unified efficacy assessment criteria for natural products against UC, incorporating multidimensional indicators including macroscopic phenotypes, histopathology, molecular biology, gut microbiota, and intestinal barrier function. Employ multi-omics technologies (genomics, transcriptomics, proteomics, metabolomics) to comprehensively decipher the molecular networks through which natural products regulate MAPK and interact with pathways such as NF-κB and Nrf2, elucidating their multi-target synergistic mechanisms. Third, enhance safety evaluation and quality control: Conduct systematic long-term toxicity studies, assess effects on hepatic and renal function, evaluate reproductive toxicity, and define safe dosage ranges for natural products. Standardize their origin, harvesting, extraction, and purification processes to establish unified quality control standards, ensuring stability and uniformity of active ingredient content. Simultaneously, investigate interactions between natural products and first-line drugs to mitigate combination therapy risks. Fourth, advance translational medicine and drug development: Enhance pharmaceutics research on natural products by improving bioavailability and targeting through delivery systems like nanocarriers, liposomes, and microspheres, or by optimizing pharmacokinetic properties via structural modification. Conduct small-scale, exploratory clinical trials to evaluate the safety and efficacy of natural products as monotherapies or in combination with conventional drugs, accumulating evidence-based medical data. Encourage multidisciplinary collaboration to integrate modern drug development technologies with traditional natural medicine research, facilitating the transformation of natural products into standardized pharmaceuticals.

In summary, natural products targeting MAPK for UC treatment demonstrate significant multi-target synergistic advantages and clinical application potential. However, current research faces challenges including models that do not closely resemble clinical settings, inconsistent evaluation systems, inadequate safety assessments, and weak translational research. Future efforts should focus on key measures such as developing precise models, establishing standardized systems, strengthening safety controls, and advancing translational research to overcome existing bottlenecks. This will fully unlock the therapeutic value of natural products, providing more efficient and safer new strategies and treatment options for the clinical management of UC.

## Glossary

UC: ulcerative colitis
IBD: inflammatory bowel disease
EGFR: epidermal growth factor receptor
OS: Oxidative stress
ROS: reactive oxygen species
MAPK: mitogen-activated protein kinase
IL-1β: Interleukin-1β
MDA: Malondialdehyde
PI3K: Phosphatidylinositol 3-kinase
SOD: Superoxidedismutase
GSH: Glutathione
SOD: Superoxidedismutase
MDA: Malondialdehyde
Nrf2: NFE2-related factor 2
NF-κB: Nuclear factor kappa-B
TNF-α: tumor necrosis factor-α
DCs: Dendritic Cells
Bax: Bcl2-Associated X
Bcl-2: B-cell lymphoma-2
CGA: Chlorogenic acid
EA: Ellagic acid
BCA: Biochanin A
CY: Carthamin yellow
KSCI: Kushenol I
LDC: Linderanine C
BI: Bilobalide
OAG: Oleanolic acid 28-O-β-D-glucopyranoside
SCFA: short-chain fatty acid
AB4: Anemoside B4
PA: Paeoniflorin
PE: Pedunculoside
AS: Asiaticoside
NCF: Nuciferine
BBR: Berberine
MBP: Mesona chinensis Benth polysaccharides
PPBP: Pyrus pashia Buch polysaccharide
ZOP: Zingiber officinale polysaccharide
CNLP: Polysaccharides of C. nudiflora Hook
SA: Shikimic acid
NADD: N-Acetyldopamine Dimer
7-HC: 7-hydroxycoumarin
AR: Amauroderma rugosum
EEAR: Extract of Atractylodis Rhizoma
CUP: Citrus unshiu peel water extract
AFE: Anneslea fragrans extract
FEC: Flower extract of Caragana sinica
SYR: Syringaresinol
PHI: Phillygenin
ALT: Atractylodin
CA: Chebulagic acid
